# Arctic Fox-Derived *Lactiplantibacillus plantarum* J3 Alleviates Colitis in Mice in Association with Strengthening of the Intestinal Barrier and Reshaping of the Intestinal Flora

**DOI:** 10.3390/life16081355

**Published:** 2026-08-18

**Authors:** Yiwen Sun, Jing Lv, Xiangyu Meng, Yanqiu Sun, Hualin Fu, Wei Xu, Zhiheng Du

**Affiliations:** College of Animal Sciences and Technology, Northeast Agricultural University, Harbin 150030, China; 15135520115@163.com (Y.S.); lvjing@neau.edu.cn (J.L.); mengxy0706@163.com (X.M.); s2751908995@163.com (Y.S.); 15069496688@163.com (H.F.); 15937335908@163.com (W.X.)

**Keywords:** arctic fox, *Lactiplantibacillus plantarum*, intestinal microflora, intestinal barrier, mouse model, colitis

## Abstract

Probiotic interventions for inflammatory bowel disease (IBD) have become a hot research topic in this field. However, no previous studies have investigated whether fox-derived probiotics exert therapeutic effects against colitis. The present investigation evaluated the effects of *Lactiplantibacillus plantarum* (syn. *Lactobacillus plantarum*) J3, sourced from the intestines of healthy Arctic foxes, on colitis. In an in vitro LPS-stimulated Caco-2 cell model, strain J3 exhibited prominent anti-inflammatory and antioxidant capacities, indicating potential to preserve intestinal barrier integrity. Furthermore, in an in vivo DSS-induced colitis mouse model, oral J3 supplementation markedly alleviated body weight loss, lowered disease activity index (DAI), and ameliorated histopathological lesions in colon tissues. Mechanistic analyses revealed that the J3 group displayed significantly decreased colonic levels of TNF-α, IL-1β, IL-6, MPO, PGE_2_, along with downregulated *COX-2* mRNA expression (*p* < 0.05). By contrast, the levels of IL-10, TJ protein mRNA, and *MUC2* mRNA were markedly upregulated, whereas serum LPS and D-Lac concentrations were significantly reduced (*p* < 0.05). In an exploratory analysis of 16S rDNA and short-chain fatty acids (SCFAs) in a subset of mice, J3 treatment showed a trend toward colonic microbial remodeling, accompanied by a significant increase in short-chain fatty acid production (*p* < 0.05). Overall, these findings indicate that *Lactiplantibacillus plantarum* J3 derived from foxes may alleviate DSS-induced colitis in mice, providing a basis for future translational research.

## 1. Introduction

Posing a considerable health challenge, inflammatory bowel disease (IBD) extends from humans to companion animal species like dogs and cats [1]. Ulcerative colitis (UC), a major clinical subtype of IBD, mainly targets the colon and rectum and presents with clinical manifestations including abdominal pain, hematochezia, and body weight loss [2,3]. Nevertheless, current clinical interventions for IBD remain suboptimal. Conventional therapeutic regimens, such as aminosalicylates, corticosteroids, and fecal microbiota transplantation [4,5], frequently lead to primary or secondary treatment failure, accompanied by safety risks, multiple side effects, high medical costs, and the absence of accurate predictive biomarkers [6,7]. Therefore, identifying safe, effective, and cost-effective interventions is key to addressing the current challenges in IBD treatment.

Probiotics have attracted extensive research attention owing to their capacities to maintain a balanced intestinal environment [8], emerging as a core research direction for IBD therapeutic development [9,10]. *Lactiplantibacillus plantarum* has been widely investigated for its favorable safety profile and versatile probiotic properties. This species exerts multiple gut-beneficial effects by secreting organic acids to reduce luminal pH. Moreover, *Lactiplantibacillus plantarum* exhibits robust intestinal colonization capacity: after attaching to the intestinal wall via adhesins, it can colonize and exert long-term probiotic effects [11]. Certain strains of *Lactiplantibacillus plantarum* also secrete antimicrobial compounds (such as bacteriocins) to directly inhibit pathogenic microorganisms [12] and modulate the host’s gut microbiota [13]. In the context of UC, the barrier-protective effect exerted by this strain is typically associated with elevated levels of tight junction (TJ) proteins and mucins [14]. Beyond this, *Lactiplantibacillus plantarum* has also demonstrated significant efficacy in suppressing inflammatory signaling pathways and regulating cytokine balance [15].

As strict carnivores, Arctic foxes harbor a unique intestinal ecosystem that may host functionally distinctive probiotic strains. In addition, considering the pathological similarities between IBD and weaning stress syndrome in Arctic fox cubs [16,17,18], we hypothesized that fox-origin probiotics could alleviate IBD-associated intestinal inflammation. We identified a strain derived from the Arctic fox—*Lactiplantibacillus plantarum* J3. Whole-genome sequencing revealed that the J3 strain possesses 196 strain-specific genes, as well as five plasmids, and multiple genes involved in quorum sensing, carbohydrate metabolism, and fatty acid biosynthesis; these characteristics may be related to its gut colonization and probiotic functions. In our experiments, the J3 strain exhibited potent anti-inflammatory, antioxidant, and barrier-enhancing effects in vitro and significantly alleviated DSS-induced colitis in vivo. Our results suggest that J3 may represent a promising candidate within the emerging class of microbiota-based therapeutics for IBD.

## 2. Materials and Methods

The animal experimentation and sample collection protocols used in this study were approved by the Experimental Animal Ethics Committee of Northeast Agricultural University (No.: NEAUEC20230258). Detailed information regarding the reagents and materials is summarized in Supplementary Material File S3.

### 2.1. Strain Preparation and Probiotic Properties

#### 2.1.1. Separation and Identification

Colonic contents (1.0 g) from Arctic foxes were thoroughly homogenized with 9 mL of 0.9% NaCl, followed by a 10-fold serial dilution (10^−1^ to 10^−4^). Aliquots of each dilution were then spread onto de Man, Rogosa, and Sharpe (MRS) agar plates containing 1.5% CaCO_3_, and the plates were incubated at 37 °C for 48 h under anaerobic conditions using gas-generating sachets (AnaeroPack, Mitsubishi Gas Chemical, Tokyo, Japan) in sealed anaerobic jars (Oxoid, Basingstoke, UK). Nineteen colonies with distinct calcium-dissolving zones were selected and purified through three consecutive streaking procedures. Following the method described in Reference [19], all strains were identified as *Lactiplantibacillus plantarum* through 16S rDNA gene sequencing.

#### 2.1.2. In Vitro Screening and Genome-Wide Sequencing

Using gastrointestinal fluid tolerance (pH 3.0 saline + 3 g/L pepsin, 4 h; pH 8.0 saline + 1 g/L trypsin + 3 g/L bile salts, 3 h) and Caco-2 cell adhesion rate (approximately 1 × 10^8^ CFU/well, 2 h), strain J3 was selected from 19 isolates as the superior strain (see Supplementary Material File S1 for detailed procedures and data). Whole-genome sequencing of J3 was performed using the Illumina NovaSeq 6000 platform. The genomic safety assessment was conducted following the strategy described by Chokesajjawatee et al. [20]. Details of the assembly and annotation workflows, as well as the specific methods and results of the safety assessment, are provided in Supplementary Material File S2 (Section S2.11). J3 was stored at −80 °C in 30% glycerol-MRS.

#### 2.1.3. Formulations

Following 18 h of propagation in MRS medium at 37 °C, the J3 cells were harvested via centrifugation (8000× *g*, 10 min), rinsed twice with sterile Phosphate-Buffered Saline (PBS), and collected for further use. The resulting cell pellets were resuspended separately in sterile PBS (for mouse gavage, 1 × 10^10^ CFU/mL) or antibiotic-free Dulbecco’s Modified Eagle Medium (DMEM) (for cell experiments, 1 × 10^8^ CFU/mL). Solutions were prepared immediately before use.

### 2.2. Test Management and Sample Collection

#### 2.2.1. In Vitro Assay Using Caco-2 Cells

Caco-2 cells, obtained from the Cell Bank of the Chinese Academy of Sciences, were subsequently maintained in DMEM supplemented with 10% fetal bovine serum and 1% penicillin-streptomycin at 37 °C under 5% CO_2_. Cells from passages 25–35 were used for all experiments. Prior to the experiments, the cells were transferred to antibiotic-free DMEM and allowed to acclimate for 24 h.

A total of 48 subjects were randomly divided into four groups (*n* = 12 per group). No animals died or were removed during the experiment; all mice survived to the endpoint:

Based on the treatments applied, three groups were designated:

Control group: Cells were maintained in antibiotic-free DMEM medium under standard conditions for 24 h.

LPS group: Cells received 1 μg/mL lipopolysaccharide (LPS) from Escherichia coli O55:B5 (Sigma-Aldrich, St. Louis, MO, USA) stimulation for 24 h. The LPS concentration of 1 μg/mL was selected based on previous studies demonstrating that this concentration effectively induces inflammatory responses in Caco-2 cells without causing cytotoxicity [21,22,23,24].

J3 + LPS group: Cells were pretreated for 1 h with a J3 preparation at a final concentration of 1 × 10^8^ CFU/mL. After pretreatment, they were co-cultured with 1 μg/mL LPS for 24 h.

The supernatant and cells from each treatment group were collected separately for subsequent experiments.

#### 2.2.2. Experimental Study Using a Murine Model of DSS-Induced Colitis

Forty-eight male C57BL/6J mice, aged four weeks, were equilibrated under a duration of 1 week under standardized environmental conditions (humidity: 50 ± 10%, temperature: 22 ± 2 °C, lighting: 12 h light/dark cycle).

A total of 48 subjects underwent randomization into four experimental cohorts:

Control group: Days 1–21: 0.2 mL of sterile PBS administered via oral gavage daily.

DSS group: Days 1–7: 3% DSS (36–50 kDa) was provided ad libitum in the drinking water. Days 8–21: 0.2 mL of sterile PBS was administered via oral gavage daily.

J3 + DSS group: Days 1–7: Modeling with DSS; Days 8–21: Daily intragastric administration of J3 (0.2 mL, 1 × 10^10^ CFU/mL) was conducted. This dose was selected based on the effective dose of *Lactiplantibacillus plantarum* strains previously reported in DSS-induced colitis models [13,25].

Positive drug control group: Days 1–7: Modeling with DSS; Days 8–21: Sulfasalazine (SASP) was administered orally at 200 mg/kg per day.

In the mouse experiment, mice were weighed daily, and their droppings’ texture and rectal bleeding in the feces were observed, from which a disease activity index (DAI) was calculated according to the criteria listed in Table 1. The DAI scores were assessed independently by two observers who were unaware of the experimental group assignments, and the average score was used for statistical analysis. Upon completion of the study, the mice were deeply sedated and euthanized according to the method described in [26], and samples were collected. Colon length was recorded. Serum was collected, with tissue aliquots and colon contents snap-frozen at –80 °C, and additional colonic segments fixed for histopathological examination.

### 2.3. Histopathological Analysis of Mouse Colon Tissue

Fixed colon tissues were paraffin-embedded and microtomed (7 μm). Sections were then subjected to Hematoxylin and Eosin (H&E) staining to observe morphological changes, and with Alcian Blue-Periodic Acid-Schiff (AB-PAS) (pH 2.5) to count goblet cells. From each section, 5 fields of view were randomly selected at a magnification of 200×. Using ImageJ software (version 1.53), the total mucosal area (mm^2^) was measured in each field of view, and the number of AB-PAS-positive cells was counted. The results were expressed as the number of goblet cells per mm^2^ of mucosa, and the mean of the 5 fields of view was taken as the final value for that sample.

### 2.4. Enzyme-Linked Immunosorbent Assay (ELISA)

Caco-2 cell supernatant was assayed for tumor necrosis factor-α (TNF-α), interleukin-6 (IL-6), interleukin-1β (IL-1β), and interleukin-10 (IL-10) using the kit as per the manufacturer’s protocol.

With this kit, and per the manufacturer’s protocols, quantify myeloperoxidase (MPO) catalytic activity, along with the concentrations of Prostaglandin E_2_ (PGE_2_), TNF-α, IL-1β, IL-6, and IL-10 in mouse colon tissues. Circulating LPS and D-lactate abundances in murine serum were also determined simultaneously.

### 2.5. Profiling Reactive Oxygen Species (ROS) and Mitochondrial Functional Traits in Caco-2 Cells

After the Caco-2 cells were administered the interventions specified in Section 2.2.1, they were rinsed using PBS. The fluorescent probe 2′,7′-dichlorodihydrofluorescein diacetate (DCFH-DA) was prepared to 10 μM in phenol red- and serum-deprived DMEM, followed by incubation with cells under dark conditions at 37 °C and 5% CO_2_ over a 30 min period. After washing three times, the signal intensity of intracellular fluorescence was acquired using 485 nm excitation and 528 nm emission filters to quantify ROS. Under the same conditions, incubate with 5 μM MitoSOX Red and 10 μg/mL Rhodamine 123 (Rh-123), respectively. Upon washing, acquire fluorescent signals using dual-wavelength settings: 510 nm (excitation)/580 nm (emission) and 507 nm (excitation)/529 nm (emission), which enables detection of mitochondrial superoxide radicals and the membrane potential (ΔΨm). All fluorescence intensities were measured using a SpectraMax iD3 microplate reader, and relative fluorescence intensities were calculated with the control group as the reference.

### 2.6. Determination of Trans-Epithelial Resistance (TEER) in Caco-2 Cells

Trypsinized Caco-2 cells were plated onto 0.4 μm-pore Transwell chambers (Corning, New York, NY, USA) at 1 × 10^5^ cells per well. Incubation proceeded at 37 °C with 5% carbon dioxide, and fresh culture medium was replenished on alternate days. Periodic recordings of transepithelial electrical resistance (TEER) were performed via a Millicell ERS-2 epithelial volt-ohm meter (Millipore, Billerica, MA, USA) until readings plateaued above 500 Ω·cm^2^, an indicator of intact tight monolayer formation. Results were calculated using the following formula:

(1) $$\mathrm{TEER}\left(\Omega \cdot {\mathrm{cm}}^{2}\right)=\left(\mathrm{sample}\mathrm{resistance}-\mathrm{blank}\mathrm{resistance}\right)\times \mathrm{effective}\mathrm{membrane}\mathrm{area}\left(1.13\;{\mathrm{cm}}^{2}\right).$$

where the blank resistance is the resistance value of the Transwell membrane without cells (approximately 220 Ω).

### 2.7. Gene Expression Analysis (qPCR)

Total RNA was extracted from Caco-2 cell cultures using TRIzol, with RNase-free DNase I added during the extraction process to remove residual genomic DNA contamination. The RNA was quantified using a NanoDrop 2000 spectrophotometer (Thermo Fisher Scientific, Waltham, MA, USA); the A260/A280 ratios for all samples ranged from 1.8 to 2.0, indicating good purity. RNA integrity was assessed using an Agilent 2100 Bioanalyzer (Agilent Technologies, Santa Clara, CA, USA). The RNA Integrity Number (RIN) values for all samples were greater than 8.0, indicating that the RNA showed no significant degradation. The resulting total RNA was then reverse-transcribed into cDNA using the PrimeScript™ RT Kit. Mouse colon tissue samples were homogenized under liquid nitrogen conditions, and total RNA was extracted following the same procedure. After DNase I treatment and verification of the A260/A280 ratio and RIN value to confirm quality, cDNA synthesis was performed using the GoScript™ reverse transcription kit. qPCR analysis was conducted on the ABI 7500 system. Transcription levels of the candidate genes were normalized using the independent internal reference genes GAPDH and β-actin. Relative expression profiles were calculated using the 2^−ΔΔCt^ method based on the cycle threshold comparison; raw data were calibrated against control samples. The nucleotide sequences of all target amplification primers are listed in Supplementary Material File S3.

### 2.8. 16S rDNA Gene Amplicon Profiling of Murine Colon Microbial Contents

Illumina MiSeq high-throughput sequencing was adopted to characterize microbial communities recovered from mouse colonic luminal specimens. The main workflow included: genomic DNA was purified using the QIAamp DNA Kit; amplification of the 16S rDNA V3–V4 region using primers 338F/806R; and 2 × 300 bp paired-end sequencing following purification and quantification. Raw sequencing data were analyzed using the QIIME 2 platform (version 2024.2). The DADA2 plugin was used to perform quality filtering, denoising, merging, and chimera removal on paired-end sequences, generating an amplicon sequence variant (ASV) feature table. For species annotation, a pretrained Naive Bayes classifier (based on the Silva 138 database, with 99% of OTU reference sequences) was used to classify representative ASV sequences. α-diversity indices (Chao1, Shannon, Simpson) and β-diversity (Principal Coordinate Analysis (PCoA) based on Bray–Curtis distance) were calculated using the normalized ASV feature table. 16S sequencing was performed on an exploratory subset of 3 mice per group. Due to the small sample size (*n* = 3), microbial abundance data are presented descriptively only, and no formal hypothesis tests were conducted. Detailed statistical test results (Mann–Whitney U test and FDR-corrected *p*-values) are provided in the Supplementary Materials File S4. The Linear Discriminant Analysis Effect Size (LEfSe) method (Linear Discriminant Analysis (LDA) threshold > 2.0) was performed on the Meiji Bio Cloud Platform (https://cloud.majorbio.com) to identify taxonomic biomarkers capable of distinguishing between the study groups.

### 2.9. Determination of Short-Chain Fatty Acids in Mice

Weigh 0.80 ± 0.01 g of mouse colon contents and homogenize them in sterile PBS to prepare a 10% (*w*/*v*) homogenate suspension. The 500-μL slurry was mixed with 100 μL of crotonic acid solution as an internal standard and stored at −30 °C for 24 h. After thawing, the mixture was centrifuged at 4 °C and 8000× *g* for 3 min. Post filtration of the collected liquid phase, the specimen was loaded onto the apparatus for measurement.

Chromatographic partitioning was accomplished with an Agilent DB-FFAP capillary tubing column (30 m × 0.25 mm × 0.25 μm) mounted on an Agilent 7890B gas chromatography unit (Santa Clara, CA, USA) fitted with a flame ionization sensing module calibrated to 250 °C. Comprehensive inlet, carrier gas, and oven thermal gradient specifications are documented in Supplementary Material File S3. Short-Chain Fatty Acids (SCFAs) concentrations were calculated using calibration curves.

For SCFAs data, comparisons among the four groups were performed using the Kruskal–Wallis test, followed by Dunn’s post hoc test, with Benjamini–Hochberg FDR correction for multiple comparisons; a corrected *p*-value < 0.05 was considered statistically significant. The SCFAs analysis data were derived from an exploratory subset (3 mice per group), and the results should be considered preliminary findings.

### 2.10. Statistical Analysis

All statistical computations were executed via the SPSS 23.0 statistical package. Normality distribution of datasets was assessed through the Shapiro–Wilk procedure, whereas Levene’s assay served for the validation of variance homogeneity. Prerequisites for normal distribution and equal variance among study cohorts were fully satisfied across all measured data. Inter-group disparity evaluation was carried out via one-way ANOVA, and Tukey’s HSD multiple comparison tests were executed as subsequent validation steps. A *p*-value cutoff of less than 0.05 was adopted to identify statistically relevant discrepancies. In vitro experiments were performed in three independent replicates, each with three replicate wells. In animal experiments, each mouse was considered an independent biological replicate (*n* = 12 per group). See the captions for each figure for the specific sample sizes for each indicator, and three technical replicates were performed for each sample, with the mean calculated, and resultant readings are reported as mean ± standard deviation (SD).

## 3. Results

### 3.1. LPS-Induced Inflammation and Oxidative Stress in Enterocyte Monolayers

**Figure 1:**
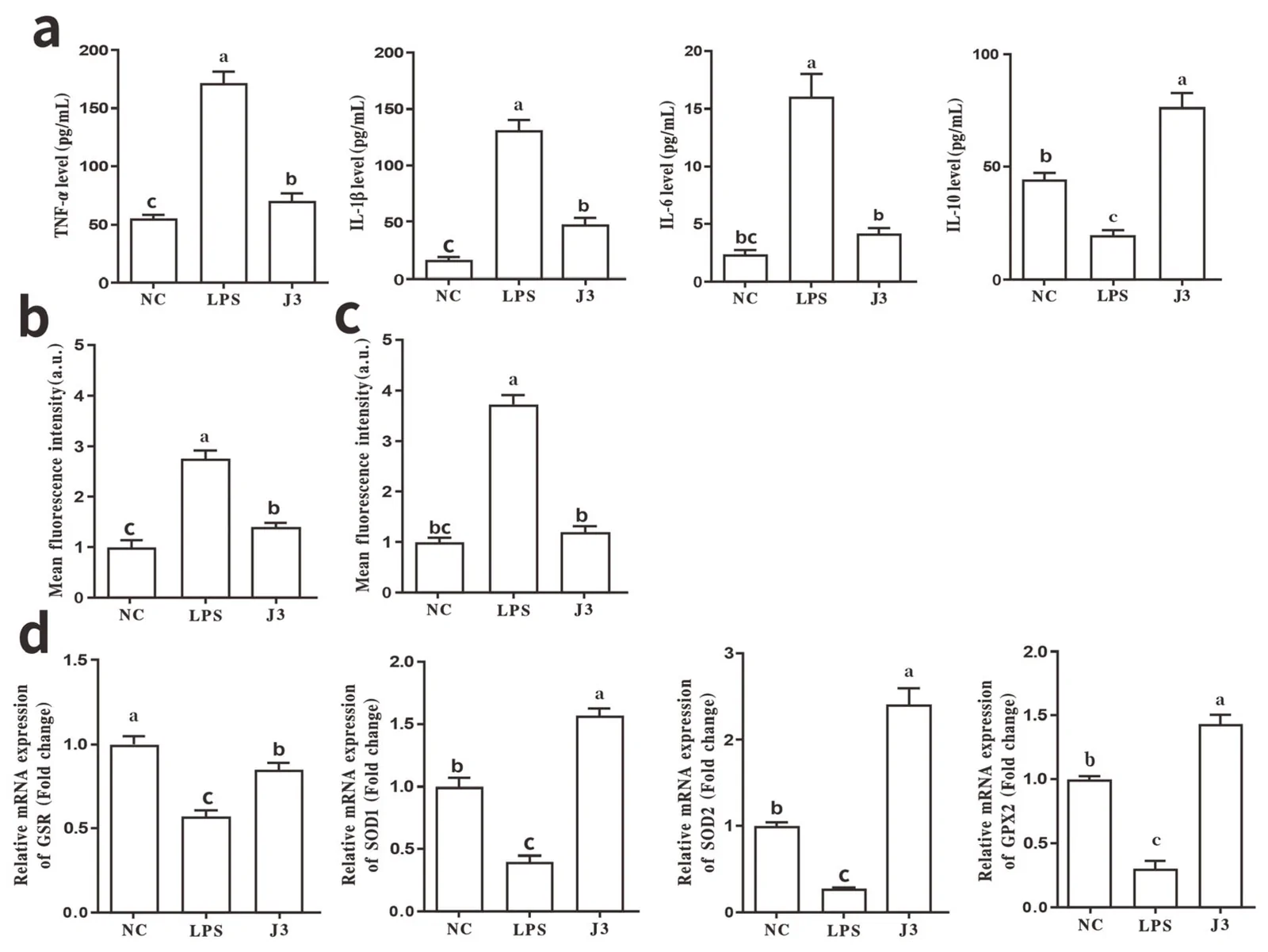
*Lactiplantibacillus plantarum* J3 intervention mitigates LPS-triggered inflammatory and oxidative responses in polarized Caco-2 enterocytes: (**a**) Concentrations of pro-inflammatory mediators (TNF-α, IL-1β, IL-6, and IL-10); (**b**) Concentrations of ROS; (**c**) Concentrations of mitochondrial superoxide; (**d**) mRNA expression levels of antioxidant enzymes (*GSR*, *SOD1*, *SOD2*, and *GPX2*). Measurable continuous readouts are displayed in the form of mean ± SD (*n* = 3). Disparate letter annotations (a, b, c) mark any statistically meaningful variance across experimental cohorts (*p* < 0.05).

The cytoprotective potential of strain J3 was evaluated in an LPS-stimulated model by assessing the cytokine milieu. Caco-2 epithelial monolayers challenged with LPS showed significantly increased levels of pro-inflammatory cytokines (TNF-α, IL-1β, IL-6) versus blank cellular cultures. (*p* < 0.05, Figure 1a). *Lactiplantibacillus plantarum* J3 administration brought about remarkable downregulation of such inflammatory effector contents and facilitated elevated IL-10 secretion (*p* < 0.05). Strain J3 treatment significantly reduced the overall intracellular ROS levels and mitochondrial superoxide species, alongside elevating transcript abundance of antioxidant enzymatic genes including Glutathione Reductase (*GSR*), Superoxide Dismutase 1 (*SOD1*), Superoxide Dismutase 2 (*SOD2*), and Glutathione Peroxidase 2 (*GPX2*) (*p* < 0.05, Figure 1b–d).

### 3.2. Caco-2 Cell Intestinal Barrier Function

**Figure 2:**
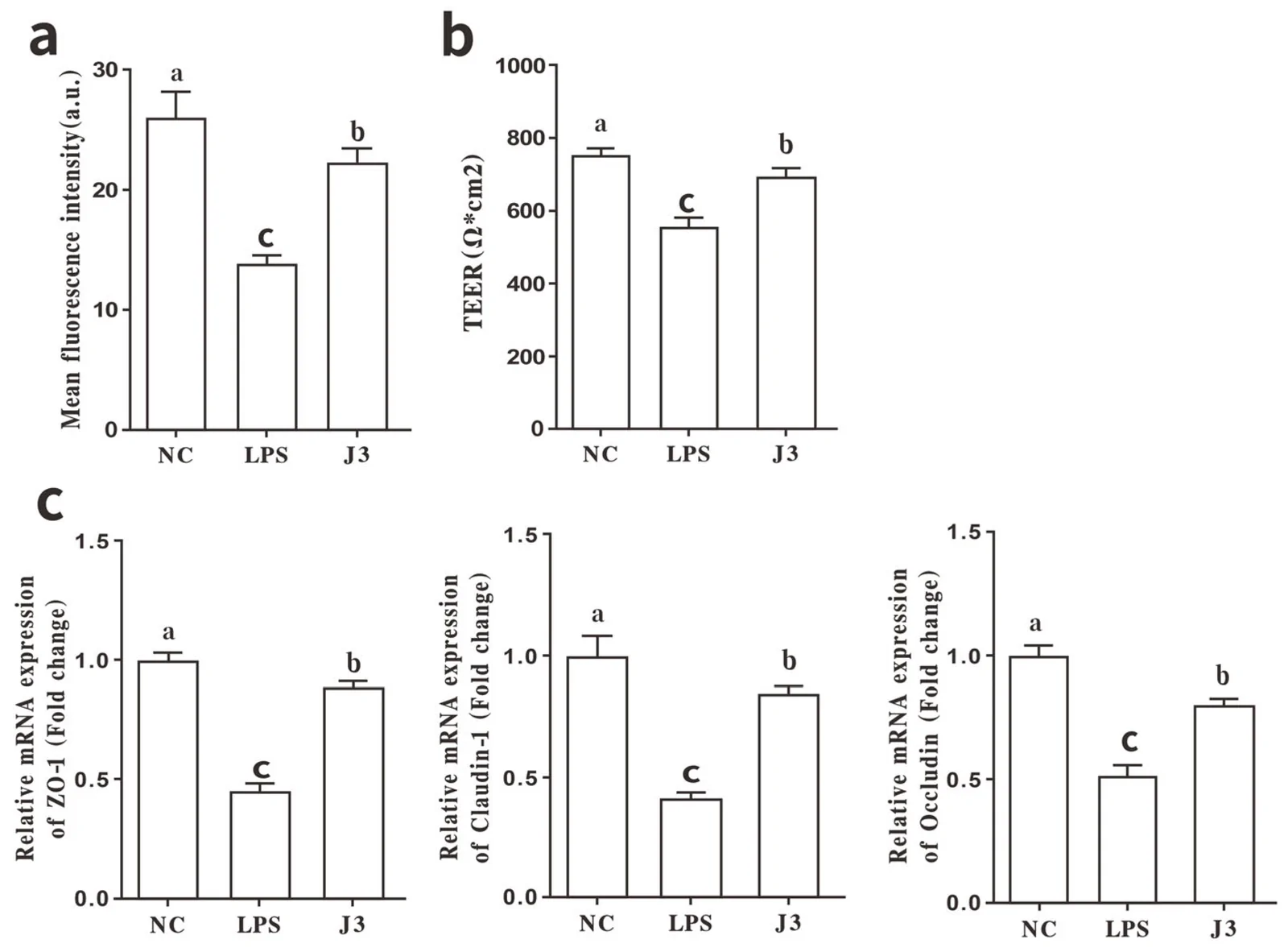
Impact of *Lactiplantibacillus plantarum* J3 on LPS-Induced gut barrier competence in Caco-2 Cell Models: (**a**) Mitochondrial membrane potential; (**b**) TEER; (**c**) mRNA expression levels of tight junction genes (*ZO-1*, *Occludin*, and *Claudin-1*). Measurable continuous readouts are displayed in the form of mean ± SD (*n* = 3). Disparate letter annotations (a, b, c) mark any statistically meaningful variance across experimental cohorts (*p* < 0.05).

Disruption of mitochondrial membrane potential impairs cellular energy production, induces oxidative stress, and compromises cellular health [27]. UC also causes mitochondrial dysfunction in the enteric mucosa. The integrity and permeability of the epithelial barrier are reflected by the cell membrane potential [28]. TEER and mitochondrial membrane potential were evaluated in Caco-2 cell cultures. Compared with untreated cells, Caco-2 monolayers subjected to LPS treatment showed a significant decrease in mitochondrial membrane potential (*p* < 0.05, Figure 2a). In contrast, treatment with live J3 significantly increased mitochondrial membrane potential (*p* < 0.05). Coincident with this, LPS exposure caused a significant decrease in transepithelial electrical resistance metrics (*p* < 0.05, Figure 2b), whereas J3 conditioning produced prominent TEER level gains over the LPS group (*p* < 0.05). Further analysis of tight junction protein mRNA expression revealed that J3 treatment reversed the LPS-induced downregulation of TJ-related genes. *Zonula Occludens-1* (*ZO-1*), *Occludin*, and *Claudin-1* mRNA levels were significantly increased following J3 exposure in comparison to the LPS group (*p* < 0.05, Figure 2c), reaching levels comparable to those of the control group.

### 3.3. Pathological Manifestations of DSS Colitis in a Murine Model

**Figure 3:**
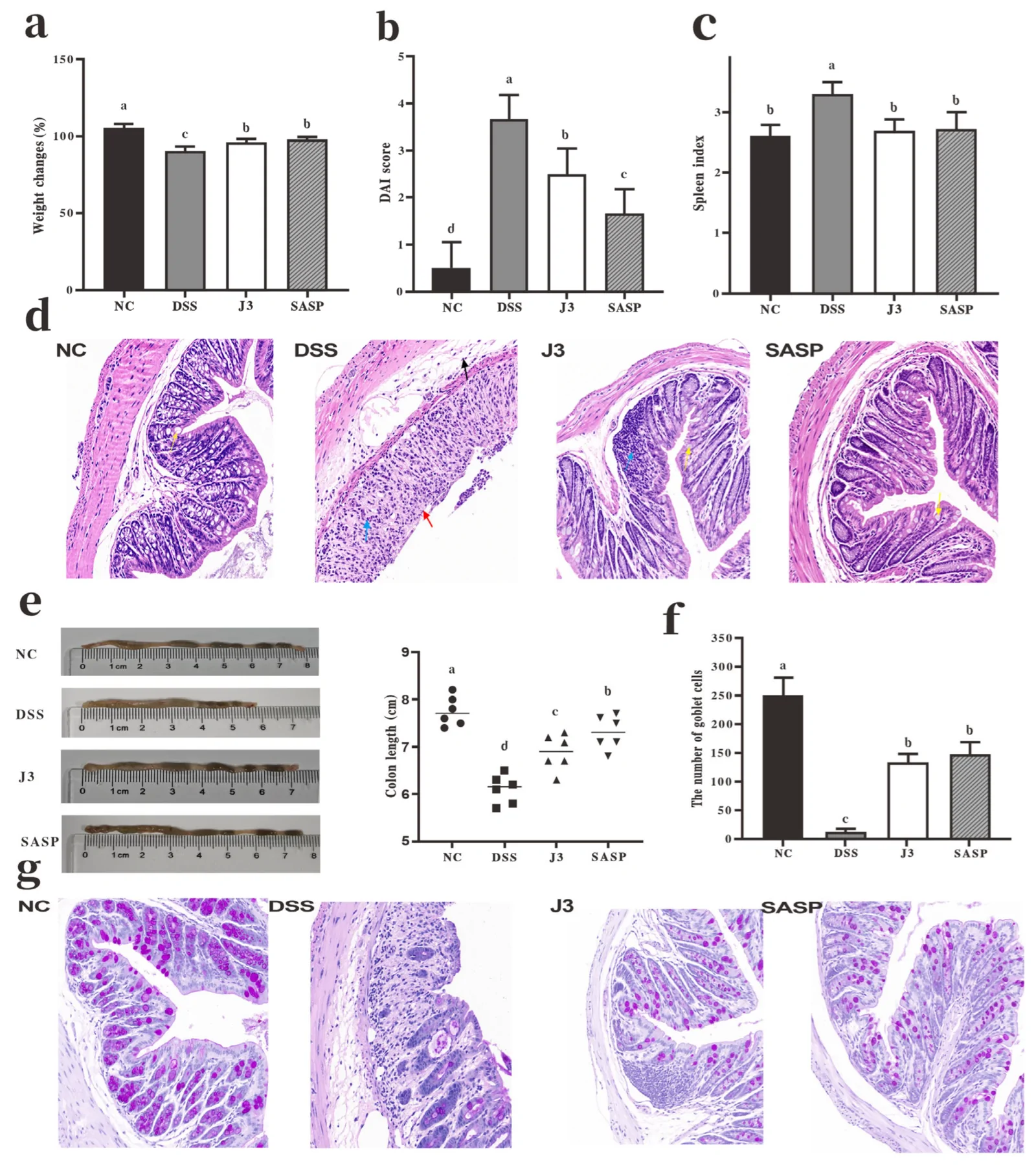
Regulatory Outcomes of J3 over Clinical Features within DSS-Caused Murine Colitis. NC: Control group; DSS: DSS group; J3: J3 group; SASP: SASP group (same below): (**a**) Variation in body mass; (**b**) Colon tissue DAI score; (**c**) Spleen index; (**d**) Colon tissue histopathology H&E staining (200×); (**e**) Quantitative analysis of colon length; (**f**) Quantification of goblet cells within colonic tissue; (**g**) AB-PAS histochemistry was applied to murine colonic specimens and examined (200×). Measurable continuous readouts are displayed in the form of mean ± SD (*n* = 12). Disparate letter annotations (a, b, c, d) mark any statistically meaningful variance across experimental cohorts (*p* < 0.05).

J3 treatment significantly alleviated the colitis phenotype induced by DSS in murine models. DAI was used to monitor colitis progression, while the spleen coefficient served as a marker of systemic inflammation and immune activation [29]. Colonic shortening is a characteristic feature of colitis [30]. J3 administration mitigated weight loss in DSS-treated mice, with effects comparable to those of SASP (*p* > 0.05). This treatment markedly lowered the DAI, attenuated the elevation of the spleen index, and attenuated colon shortening (*p* < 0.05; Figure 3a–e). Histopathological examination of the colon (Figure 3d) showed that the control group had intact structure, abundant goblet cells (yellow arrows), and no inflammatory infiltration or submucosal edema. In contrast, the DSS group exhibited severe mucosal damage, with complete epithelial detachment and exposure of the lamina propria (red arrows), destruction of crypts, widespread infiltration of inflammatory cells and fibroblasts (blue arrows), submucosal edema accompanied by mild inflammatory cell infiltration (black arrows), and a significant reduction in goblet cells. The J3 group showed neatly arranged epithelial cells, partial restoration of crypt structure, only focal inflammatory cell infiltration (blue arrows), a mild reduction in goblet cells (yellow arrows), and no submucosal edema or inflammation. The SASP group exhibited near-normal colonic histological features, with an intact epithelium, abundant goblet cells (yellow arrows), and no inflammatory infiltration or submucosal edema. Intestinal inflammation reduces the number of goblet cells and inhibits their secretion of acidic mucins, thereby impairing the mucus layer [31,32]. The number of goblet cells was significantly reduced in the DSS group (*p* < 0.05, Figure 3f,g). The number of goblet cells increased in both the J3 and SASP groups (*p* < 0.05). There was no significant difference in treatment efficacy between the J3 and SASP groups (*p* > 0.05).

### 3.4. Inflammatory Response in Mouse Colon Tissue

**Figure 4:**
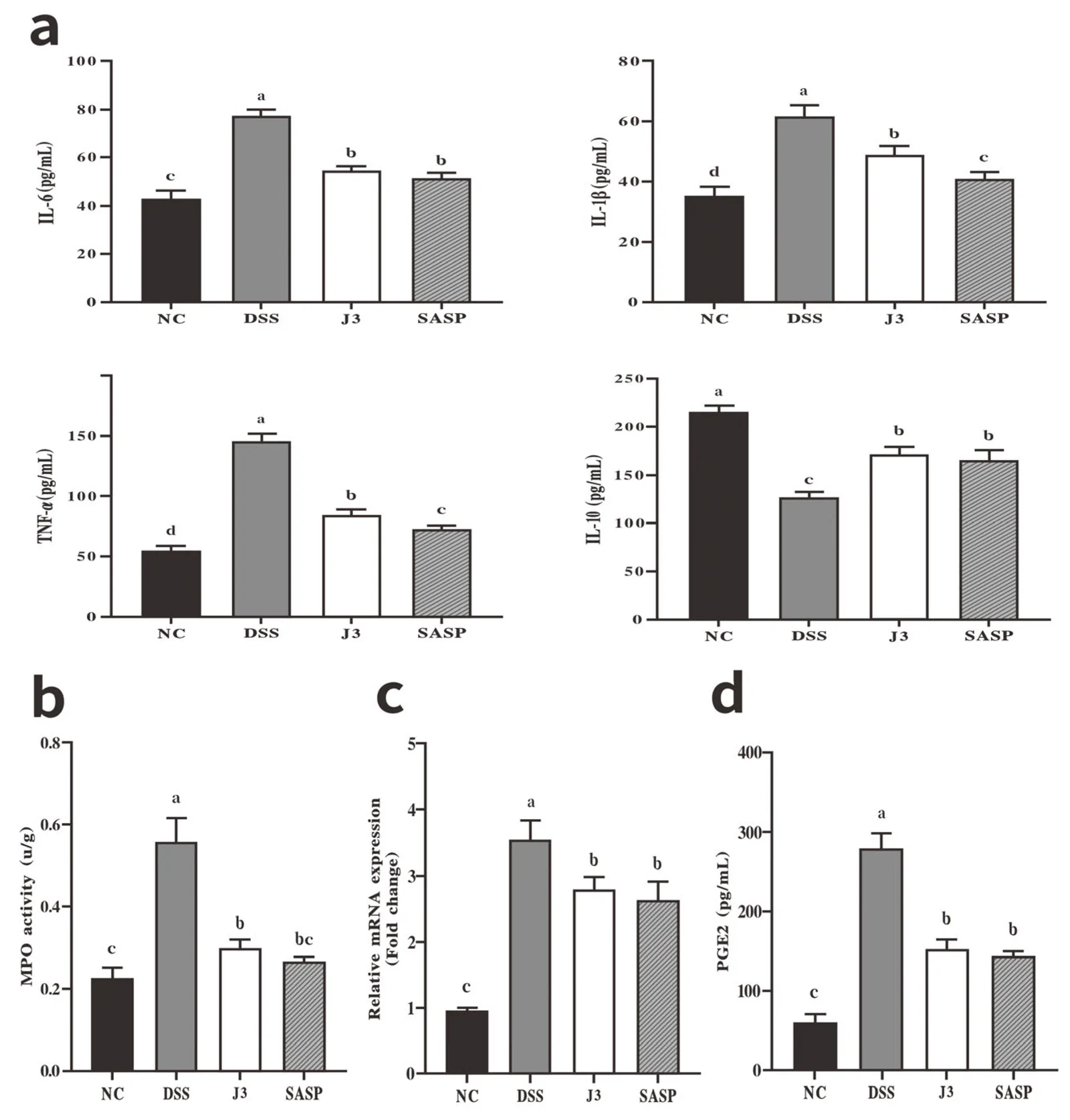
Regulatory action of J3 over DSS-elicited colonic mucosal inflammation in mouse specimens: (**a**) Concentrations of pro-inflammatory mediators (TNF-α, IL-1β, IL-6, and IL-10); (**b**) MPO catalytic activity values; (**c**) *COX-2* mRNA expression levels; (**d**) PGE_2_ tissue burdens. Measurable continuous readouts are displayed in the form of mean ± SD (*n* = 6). Disparate letter annotations (a, b, c, d) mark any statistically meaningful variance across experimental cohorts (*p* < 0.05).

Compared with the DSS group, both J3 and SASP caused significant decreases in the detected contents of MPO, PGE_2_, IL-1β, IL-6, and TNF-α (*p* < 0.05; Figure 4a,b,d), while IL-10 levels were markedly elevated (*p* < 0.05). No significant difference between groups was found in the determined quantities of PGE_2_, IL-6, or IL-10 between J3- and SASP-treated mice (*p* > 0.05). *Cyclooxygenase-2* (*COX-2*) mRNA levels were decreased—the key rate-limiting catalyst of PGE2 generation [33]—in the J3 or SASP groups. (*p* < 0.05, Figure 4c).

### 3.5. Intestinal Barrier Function in Mice

**Figure 5:**
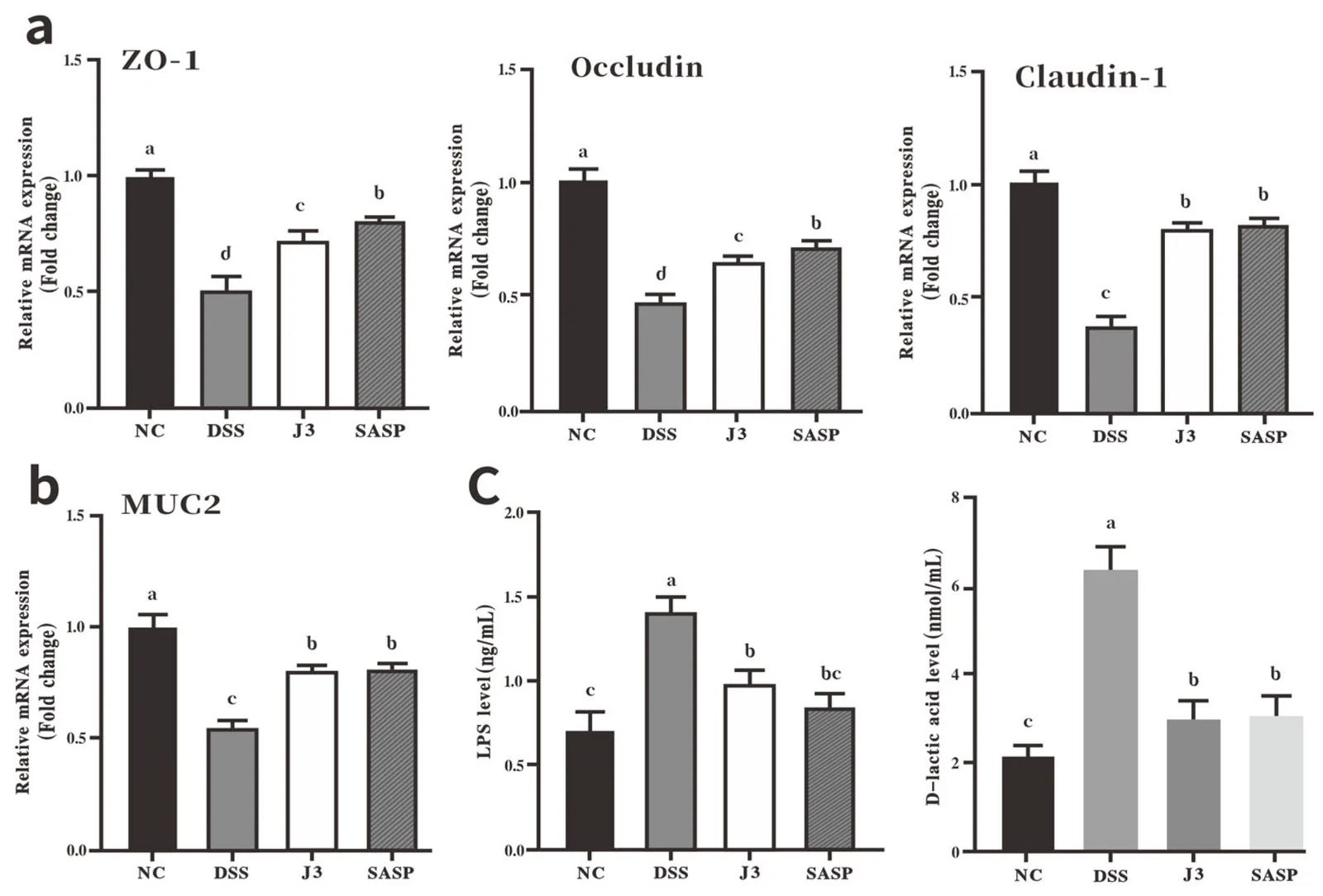
Impact of J3 on Mucosal Barrier Competence in Mice Subjected to DSS-Provoked Mucosal Damage: (**a**) TJ mRNA expression levels (*ZO-1*, *Occludin*, and *Claudin-1*); (**b**) *MUC2* mRNA expression levels; (**c**) Serum component levels (LPS and D-lactic acid). Measurable continuous readouts are displayed in the form of mean ± SD (*n* = 6). Disparate letter annotations (a, b, c, d) mark any statistically meaningful variance across experimental cohorts (*p* < 0.05).

mRNA levels of *Mucin 2* (*MUC2*) and TJ proteins (*ZO-1*, *Occludin*, *Claudin-1*) in colonic mucosa were analyzed to assess intestinal barrier function. J3 treatment significantly increased mRNA levels for all four genes (*p* < 0.05, Figure 5a,b). *MUC2* and *Claudin-1* expression levels were equivalent between the J3 and SASP groups (*p* > 0.05). In line with the above observation, serum LPS levels and D-lactate within the J3 group cohorts were significantly reduced compared with the DSS group (*p* < 0.05, Figure 5c), further confirming the improvement in intestinal barrier function.

### 3.6. SCFAs Production via Gut Microbiota Modulation in Mice

**Figure 6:**
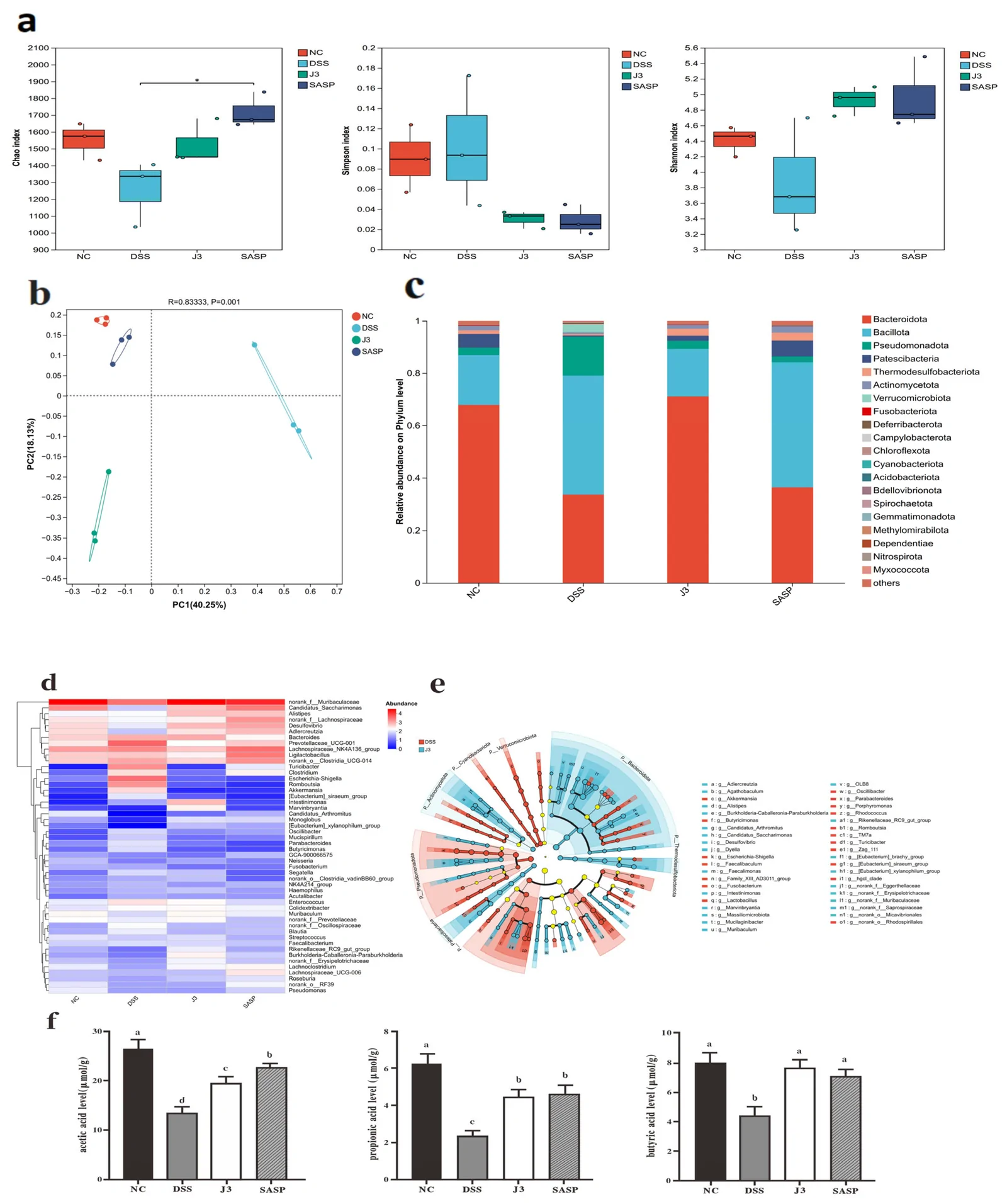
Impact of J3 on gut microbiota composition and SCFAs production in mice subjected to DSS-induced colitis: (**a**) Colon microbiota α-diversity indices (Chao1, Shannon, and Simpson); (**b**) Colon microbiota β-diversity (PCOA); (**c**) Species profiling bar chart at the phylum level; (**d**) Heatmap of species distribution among genera; (**e**) LEfSe cladogram of differentially abundant taxa between the DSS and J3 groups (LDA > 2.0); (**f**) Profiling of colonic SCFAs storage magnitudes (acetate, propionate, butyrate) Measurable continuous readouts are displayed in the form of mean ± SD (*n* = 3). Disparate letter annotations (a, b, c, d) mark any statistically meaningful variance across experimental cohorts (*p* < 0.05).

The results of 16S rDNA gene sequencing of the colonic microbiota showed the α-diversity indices across the groups, as shown in Figure 6a. β-diversity analysis indicated that the microbial community underwent structural reorganization (Figure 6b). At the phylum level, the J3 group exhibited higher proportions of *Bacteroidota* (mean ± SD, 71.05 ± 12.89% vs. 33.60 ± 13.58%), *Patescibacteria* (1.89 ± 1.31% vs. 0.41 ± 0.38%), and *Actinomycetota* (1.45 ± 0.29% vs. 0.73 ± 0.34%), whereas the relative abundances of *Bacillota* (18.24 ± 8.97% vs. 45.46 ± 26.05%), *Pseudomonadota* (3.10 ± 0.47% vs. 14.86 ± 14.01%), and *Verrucomicrobiota* (0.01 ± 0.01% vs. 3.12 ± 3.27%) were lower than those in the DSS group (Figure 6c). At the genus level, the J3 group exhibited lower relative abundances of *Escherichia-Shigella* (0.03 ± 0.01% vs. 12.42 ± 13.94%), *Romboutsia* (0.03 ± 0.008% vs. 7.41 ± 8.34%), *Clostridia_UCG-014* (1.22 ± 0.14% vs. 4.78 ± 4.28%), *Prevotellaceae_UCG-001* (0.74 ± 0.72% vs. 19.25 ± 15.71%) and *Lachnospiraceae_NK4A136_group* (1.91 ± 1.55% vs. 7.88 ± 9.19%), while *Candidatus_Saccharimonas* (1.86 ± 1.30% vs. 0.34 ± 0.38%), *Marvinbryantia* (0.78 ± 0.41% vs. 0.01 ± 0.003%), *Muribaculaceae* (60.96 ± 13.60% vs. 8.13 ± 2.32%), and *Desulfovibrio* (2.54 ± 2.38% vs. 0.39 ± 0.22%) were all higher than those in the DSS group (Figure 6d). LEfSe multilevel discriminant analysis (score > 2.0) confirmed that J3 treatment enriched *Actinobacteria*, *Patescibacteria*, and multiple genera within *Bacteroidota* (Figure 6e). We hypothesize that this microbial restructuring may enhance the biosynthesis of health-promoting, microbiota-derived metabolites. A targeted metabolic profiling assay was conducted to substantiate the above inference. Compared with the DSS group, J3 and SASP groups showed significantly higher colonic levels of acetate, propionate, and butyrate. (*p* < 0.05, Figure 6f).

## 4. Discussion

IBD is a multifactorial intestinal disorder [34], and the challenges in its treatment have spurred the search for alternative approaches. Probiotics enhance the host’s resistance to disease through symbiotic interactions with the gut microbiota and hold great promise [35,36]. This study found that strain J3, isolated from the intestines of healthy Arctic foxes, exhibits several unique genomic features, as revealed by whole-genome analysis, and effectively alleviated intestinal inflammation in a DSS-induced colitis model in mice. These findings provide preliminary experimental evidence for probiotic-based interventions for intestinal inflammation and suggest that J3 is a functional candidate strain warranting further evaluation; however, these results still need to be validated in additional experimental models and preclinical studies.

The Caco-2 intestinal epithelial cell line is a classic model for studying intestinal barrier function and the dynamics of bacterial adhesion and invasion [37]. Hyperactivated macrophage populations produce precursor IL-1β, whose bioactivity is unlocked through caspase-1-driven proteolytic truncation [38]; membrane receptor engagement launches NF-κB signal transduction cascades following ligand interaction, ultimately boosting the transcription of IL-6 and TNF-α [39]. J3 restored the inflammatory homeostasis of Caco-2 cells by synergistically downregulating TNF-α, IL-1β, and IL-6 while upregulating IL-10, which is consistent with the anti-inflammatory effects of Lactobacillus species [25]. It is hypothesized that J3 may inhibit the secretion of pro-inflammatory factors and enhance IL-10 production by modulating this pathway; the latter further suppresses NF-κB activation, thereby constituting a negative feedback loop [40]. Oxidative stress frequently coexists with inflammation [41]. In agreement with Liu et al. [42], J3 treatment elevated antioxidant enzyme mRNA (*SOD1*, *SOD2*, *GSR*, *GPX2*) and decreased cellular ROS and mitochondrial superoxide levels, suggesting its antioxidant potential, while TEER and TJ protein expression were used as reliable indicators of intestinal barrier integrity [43]. Augmented mitochondrial membrane polarization induced by J3 fuels adequate adenosine triphosphate (ATP) synthesis inside Caco-2 epithelial monolayers [27]. Dampened LPS-driven degradation of TJ components conferred by J3 bolsters intestinal protective barrier robustness, yielding a prominent elevation in TEER readings. This is consistent with the known role of Lactobacillus in enhancing intestinal barrier function [44]. These in vitro findings provide a mechanistic basis for the efficacy of J3 in subsequent in vivo experiments.

DSS can lead to the translocation of bacteria and antigens into the mucosa and trigger cytokine dysregulation [45]. This model is widely used for mechanistic studies of IBD [46]. The J3 group showed significant remission of DSS-induced colitis, as indicated by reduced weight loss, lowered DAI scores and spleen index, and attenuated colon shortening. Histological analysis revealed that J3 restored mucosal architecture and significantly increased goblet cell numbers. Neutrophil infiltration was largely inhibited, as shown by the significant decrease in MPO activity [47]. The J3 group significantly downregulated pro-inflammatory factors and upregulated IL-10 levels (consistent with Caco-2 results), indicating that it can promote the resolution of colonic inflammation. Furthermore, its modulatory effects on the COX-2/PGE_2_ axis suggest additional benefits in alleviating pain and local inflammation. Impaired intestinal barrier function is a hallmark pathological manifestation of IBD [17]. TJ proteins dictate the epithelial barrier’s permeability [48], while *MUC2* prevents microbial invasion and maintains mucosal homeostasis [49]. Probiotic-induced upregulation of TJ and mucin mRNA can synergistically enhance intestinal defense capabilities [8,50]. Serum LPS and D-lactic acid are commonly used markers for assessing intestinal barrier permeability. LPS is a major component of the outer membrane of Gram-negative bacteria, while D-lactic acid is a metabolic byproduct of intestinal bacteria; when the intestinal epithelial barrier is compromised, serum levels of LPS and D-lactic acid typically rise. In this study, J3 treatment significantly increased the mRNA levels of *MUC2* and tight junction proteins and reduced serum LPS and D-lactic acid levels, indicating improved intestinal barrier function and reduced systemic inflammation [51]. Probiotics can improve host health by modulating the gut microbiota [8]. IBD typically disrupts the host’s microbial diversity [52] and reduces levels of SCFAs [53]. Despite having no marked effect on α-diversity, J3 treatment restructured the gut microbial profile. As the primary degraders of dietary polysaccharides, increased abundance of the *Bacteroidota* phylum is closely associated with elevated levels of acetate, propionate, and butyrate in colonic contents [54]. As major components of SCFAs, acetic acid, propionic acid, and butyric acid can all suppress NF-κB signaling by activating G Protein-Coupled Receptor 43 (GPR43), thereby alleviating intestinal inflammation. Among these, butyric acid further enhances the anti-inflammatory effect by inhibiting histone deacetylases and activating G Protein-Coupled Receptor 109a (GPR109a), while propionic acid plays a complementary role by promoting the differentiation of regulatory T cells [55]. It should be noted, however, that under certain conditions, excessive production of acetic acid and propionic acid may be associated with adverse effects such as pro-inflammatory responses and dysregulation of immune function [56]. Nevertheless, within the scope of this experimental model, the elevation in these SCFA levels was accompanied by an improvement in colitis symptoms, suggesting that their overall effect is beneficial. The reduction in the *Verrucomicrobia* community and its representative genus, *Akkermansia*, may depend on the specific context: despite the strain’s beneficial potency in countering metabolic dysfunctions, an acute inflammatory colonic insult can drive A. muciniphila to worsen impairment through unabated proteolytic degradation of mucus layers [57,58]. The enrichment of *Candidatus Saccharimonas* further confirms the resolution of inflammation [59], while the reduction in pathogenic *E. coli* and *Shigella* (*Pseudomonadota*) indicates alleviated oxidative stress [60]. The increase in *Patescibacteria* (a group of genomic-minimalist commensal bacteria) [61] and the reversal of the *Bacillota*/*Bacteroidetes* ratio imbalance [62,63,64] may reflect improvements in the inflammatory microenvironment and the overall ecosystem following J3 treatment. Changes in the *Desulfobacterota* phylum are noteworthy. Compared with the DSS group, the relative abundance of *Desulfobacterota* was elevated in the J3 treatment group. Although this group was previously thought to be associated with mucosal toxicity due to H_2_S production [65], recent studies suggest that H_2_S at physiological concentrations may actually alleviate inflammation [66]. This may explain the reduced colonic inflammation observed in mice in the J3 treatment group compared to the DSS group; it is likely related to the fact that H_2_S in the J3 group modulates the local inflammatory environment without causing mucosal damage. At the same time, the *Pseudomonadota* phylum (a class of Gram-negative bacteria) is the source of LPS; its reduced abundance in the J3 group may have directly reduced LPS production in the gut, providing a possible microbiome-level explanation for the decrease in serum LPS levels.

One limitation of this study is that we used only male mice in our experiments, so we were unable to investigate potential sex differences. Furthermore, the causal relationship still needs to be established through mechanistic studies using germ-free animal models or fecal microbiota transplants. Second, although the DSS-induced mouse model is a classic system for studying colitis, it cannot fully replicate the complexity of human IBD; therefore, its applicability to other models and clinical studies requires further validation. In addition, this study did not include groups in which healthy cells or healthy mice were treated with J3 alone in either in vitro or in vivo experiments; therefore, the effects of J3 on the intestinal barrier and microbiota homeostasis under normal physiological conditions remain to be elucidated. Finally, both the 16S sequencing and SCFAs analysis in this study were based on small exploratory subsets (three animals per group), which have limited statistical power; therefore, the associated microbiota and metabolite data should be considered preliminary observations and require further validation in larger samples. In our discussion, we treat these findings as suggestive clues rather than causal evidence.

## 5. Conclusions

In conclusion, the Arctic fox-derived *Lactiplantibacillus plantarum* J3 alleviates DSS-induced colitis in mice; its protective effect is associated with the suppression of intestinal inflammation and the restoration of epithelial barrier integrity. In exploratory analyses, treatment with J3 was also associated with changes in the colonic microbiota composition and increased levels of SCFAs. These results suggest that this strain possesses certain probiotic potential and research value; however, its mechanism of action and limitations of application require further investigation.

## Figures and Tables

**Table 1:** Disease Activity Index (DAI) Scoring Criteria.

DAI Score	Weight Loss (%)	Fecal Characteristics	Occult Blood/Blood in Stool
0	0	Normal	Negative
1	1–5%	Normal	Negative
2	5–10%	Slightly loose	Occult blood positive
3	10–20%	Slightly loose	Occult blood positive
4	>20%	Loose	Gross bleeding

## Data Availability

The raw 16S rDNA gene sequencing data generated in this study have been submitted to the NCBI SRA database under accession number PRJNA1501693 and are publicly available. Other data may be reasonably requested from the corresponding author.

## Supplementary Materials

The following supporting information can be downloaded at: https://www.mdpi.com/article/10.3390/life16081355/s1, File S1: Figure S1: Tolerance of *Lactiplantibacillus plantarum* strains to simulated gastrointestinal fluid; Figure S2: Adherent rate of *Lactiplantibacillus plantarum* strains to Caco-2 cells; Figure S3: Effect of *Lactiplantibacillus plantarum* strains on cytokine concentrations in Caco-2 cells (TNF-α, IL-1β, IL-6 and IL-10); Figure S4: Effects of *Lactiplantibacillus plantarum* strains on oxidative stress in Caco-2 cells; Figure S5: Effect of *Lactiplantibacillus plantarum* strains on the expression levels of antioxidant enzyme mRNA in Caco-2 cells. (GSR, SOD1, SOD2 and GPX2); Figure S6: Effect of *Lactiplantibacillus plantarum* strains on the TEER in LPS-induced Caco-2 cells; Figure S7: Effect of *Lactiplantibacillus plantarum* strains on the expression levels of tight junction protein mRNA in Caco-2 cells. (ZO-1, Occludin and Claudin-1). File S2: Table S1: Preliminary genome assembly results of *L. plantarum* J3; Table S2: General feathures of *L. plantarum* J3; Table S3: Scattered repeat sequence results; Table S4: Tandem repeat sequence results; Figure S1: Genomics Islands of *L. plantarum* J3; Figure S2: GO functional annotation of *L. plantarum* J3; Figure S3: KEGG functional classification map of *L. plantarum* J3; Figure S4: Glycolysis/Gluconeogenesis of KEGG functional classification map of *L. plantarum* J3; Figure S5: Fatty acid biosynthesis of KEGG functional classification map of *L. plantarum* J3; Figure S6: COG functional annotation of *L. plantarum* J3; Figure S7: Genome drawing of *L. plantarum* J3; Figure S8: Circular plasmid maps of *L. plantarum* J3; Figure S9: Venn diagram of 16 *L. plantarum* strains by corepan analysis; Figure S10: Phylogenetic tree based on Core Gene; Figure S11: Synteny blocks of *L. plantarum* J3; Figure S12: KEGG functional classification map of *L. plantarum* J3; Table S5: Genes related to quorum sensing, carbohydrate and fatty acid metabolic pathways in *L. plantarum* J3. File S3: Table S1: Design of RT-qPCR Primers for Cell Assay; Table S2: Design of RT-qPCR Primers for Mouse Experiments; Table S3: Reagent Sources. File S4: Table S1: lefse_LDA; Table S2: mann_result at the genus level; Table S3: mann_result at the phylum level; Table S4: PCoA Principal Component Explanatory Power Table; Table S5: Table of Relative Abundance Results for Genus-Level Species; Table S6: Table of Relative Abundance Results for Species at the Phylum Level; Table S7: Table of T-Test Results for the Diversity Index.

## Abbreviations

Key Symbol Comparison Table:

Abbreviations	Full Name in English
IBD	Inflammatory Bowel Disease
LPS	Lipopolysaccharide
UC	Ulcerative Colitis
DSS	Dextran Sulfate Sodium
MRS	de Man Rogosa and Sharpe medium
PBS	Phosphate-Buffered Saline
DMEM	Dulbecco‘s Modified Eagle’s Medium
ELISA	Enzyme-Linked Immunosorbent Assay
ROS	Reactive Oxygen Species
DCFH-DA	2′7′-Dichlorodihydrofluorescein Diacetate
Rh-123	Rhodamine 123
TEER	Transepithelial Electrical Resistance
SASP	Sulfasalazine
DAI	Disease Activity Index
H&E	Hematoxylin and Eosin
AB-PAS	Alcian Blue-Periodic Acid-Schiff
MPO	Myeloperoxidase
TNF-α	Tumor Necrosis Factor-alpha
IL-1β	Interleukin-1 beta
IL-6	Interleukin-6
IL-10	Interleukin-10
PGE2	Prostaglandin E_2_
COX-2	Cyclooxygenase-2
MUC2	Mucin 2
ZO-1	Zonula Occludens-1
LDA	Linear Discriminant Analysis
LEfSe	Linear Discriminant Analysis Effect Size
SCFAs	Short-Chain Fatty Acids
PCOA	Principal Coordinate Analysis
SOD1	Superoxide Dismutase 1
SOD2	Superoxide Dismutase 2
GSR	Glutathione Reductase
GPX2	Glutathione Peroxidase 2
ATP	Adenosine Triphosphate
GPR43	G Protein-Coupled Receptor 43
GPR109a	G Protein-Coupled Receptor 109a
TJ	tight junction
SD	standard deviation
