# Supplementary material for: Arctic Fox-Derived Lactiplantibacillus plantarum J3 Alleviates Colitis in Mice in Association with Strengthening of the Intestinal Barrier and Reshaping of the Intestinal Flora
Source: Life (Basel). 2026 Aug 18;16(8):1355. doi: 10.3390/life16081355 (PMC13514317; doi:10.3390/life16081355)
Supplement: Supplementary file 1 [file life-16-01355-s001.zip › Supplementary Materials S1.pdf]

Supplementary Materials S1

1. Testing the Probiotic Properties of *Lactiplantibacillus plantarum*

1.1 Gastrointestinal tolerance of *Lactiplantibacillus plantarum*

Lactiplantibacillus plantarum must possess a certain degree of tolerance to gastrointestinal fluid stress in order to exert their probiotic functions. The gastrointestinal fluid tolerance of 19 *Lactiplantibacillus plantarum* strains was evaluated using a simulated gastrointestinal fluid tolerance test. The results are shown in Figure S1. All strains survived in the simulated gastrointestinal fluid, with survival rates ranging from 72.06% to 85.66%. Among them, *Lactiplantibacillus plantarum* strains J1, J2, J3, J4, J6 and J12 showed no significant difference compared with the control strain WCFS1 ($P > 0.05$). This indicates that *Lactiplantibacillus plantarum*J1, J2, J3, J4, J6 and J12 possess good gastrointestinal fluid tolerance.

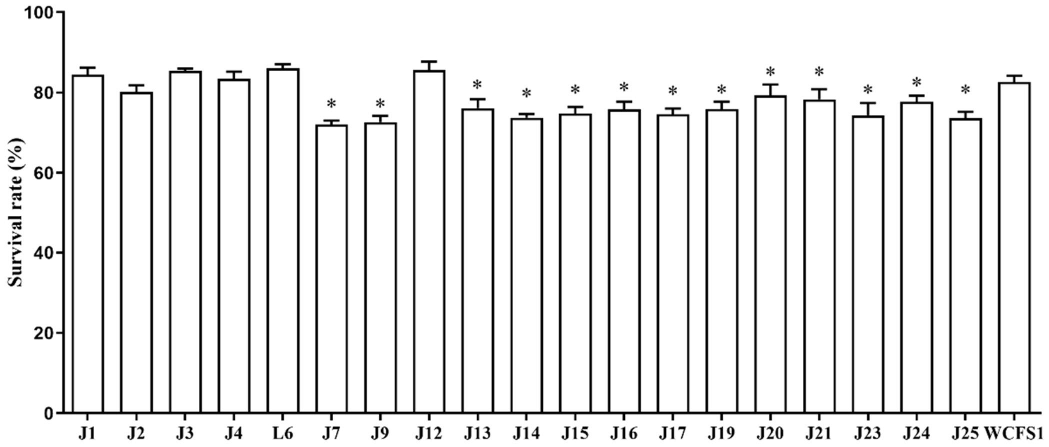

Figure S1. Tolerance of *Lactiplantibacillus plantarum* strains to simulated gastrointestinal fluid

Note: Data are mean ± SD. *represents significant difference between the test strain and *Lactiplantibacillus plantarum* WCFS1 ($P < 0.05$, one-way ANOVA with Tukey's post hoc test). The same below.

1.2 Adhesion capacity of *Lactiplantibacillus plantarum*

The adhesion rates of *Lactiplantibacillus plantarum* strains to Caco-2 cells are shown in Figure S2. All strains exhibited some degree of adhesion, with rates ranging from 3.08% to 15.39%. Among these, *Lactiplantibacillus plantarum* strains J1, J3, J4, J12 and J25 showed no significant difference from the control strain WCFS1 ($P > 0.05$). This indicates that *Lactiplantibacillus plantarum* strains J1, J3, J4, J12 and J25 possess good adhesion properties.

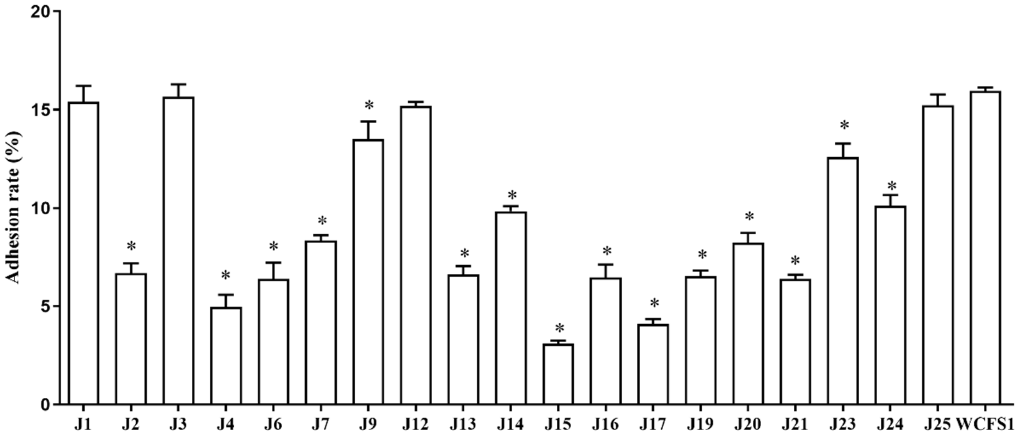

Figure S2. Adherent rate of *Lactiplantibacillus plantarum* strains to Caco-2 cells

Based on a comprehensive analysis of gastrointestinal fluid tolerance and Caco-2 cell adhesion, *Lactiplantibacillus plantarum* strains J1, J3 and J12 demonstrated generally good probiotic properties. These three strains were therefore selected for further investigation into the protective effects of *Lactiplantibacillus plantarum*on LPS-induced Caco-2 cells.

2. The effects of *Lactiplantibacillus plantarum* on LPS-induced immune modulation and oxidative stress in Caco-2 cells

2.1 The Effect of *Lactiplantibacillus plantarum* on Cytokines

As shown in Figure S3, compared with the control group (NC), LPS stimulation significantly increased the concentrations of TNF-α, IL-1β and IL-6 in Caco-2 cells, and significantly reduced the concentration of IL-10 ($P < 0.05$). *Lactiplantibacillus plantarum* strains J1, J3 and J12 all significantly reversed these changes ($P < 0.05$). In particular, the J3 treatment resulted in the lowest levels of all three pro-inflammatory factors (TNF-α, IL-1β, IL-6), with no significant difference compared to the control strain WCFS1 ($P > 0.05$); regarding IL-10, both J3 and J12 restored IL-10 levels to those comparable to WCFS1 ($P > 0.05$). Overall, J3 demonstrated the most consistent and pronounced anti-inflammatory effects.

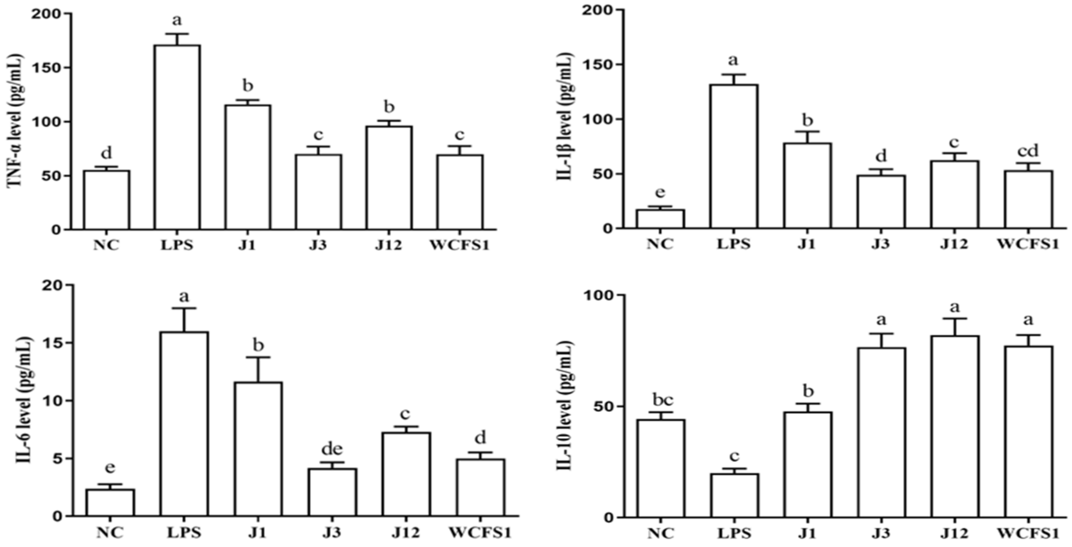

Figure S3. Effect of *Lactiplantibacillus plantarum* strains on cytokine concentrations in Caco-2 cells（TNF-α, IL-1β, IL-6 and IL-10）

Note：Data are mean ± SD. Different letters above bars indicate significant difference ($P < 0.05$, one-way ANOVA with Tukey's post hoc test). The same as below.

2.2 The effects of *Lactiplantibacillus plantarum* on cellular oxidative stress

As shown in Figure S4, compared with the control group (NC), LPS stimulation significantly increased the concentrations of ROS and mitochondrial superoxide in Caco-2 cells, whilst significantly reducing the mitochondrial membrane potential ($P < 0.05$). *Lactiplantibacillus plantarum* strains J1, J3 and J12 all significantly reversed these changes ($P < 0.05$). Among these, the J3 treatment performed best across all three parameters: following treatment with J3, ROS concentration, mitochondrial membrane potential and mitochondrial superoxide levels all returned to levels not significantly different from those of the control strain WCFS1 ($P > 0.05$), and there was also no significant difference in mitochondrial superoxide levels compared with the control group ($P > 0.05$). Overall, J3 demonstrated the most stable and prominent effect in alleviating LPS-induced oxidative stress.

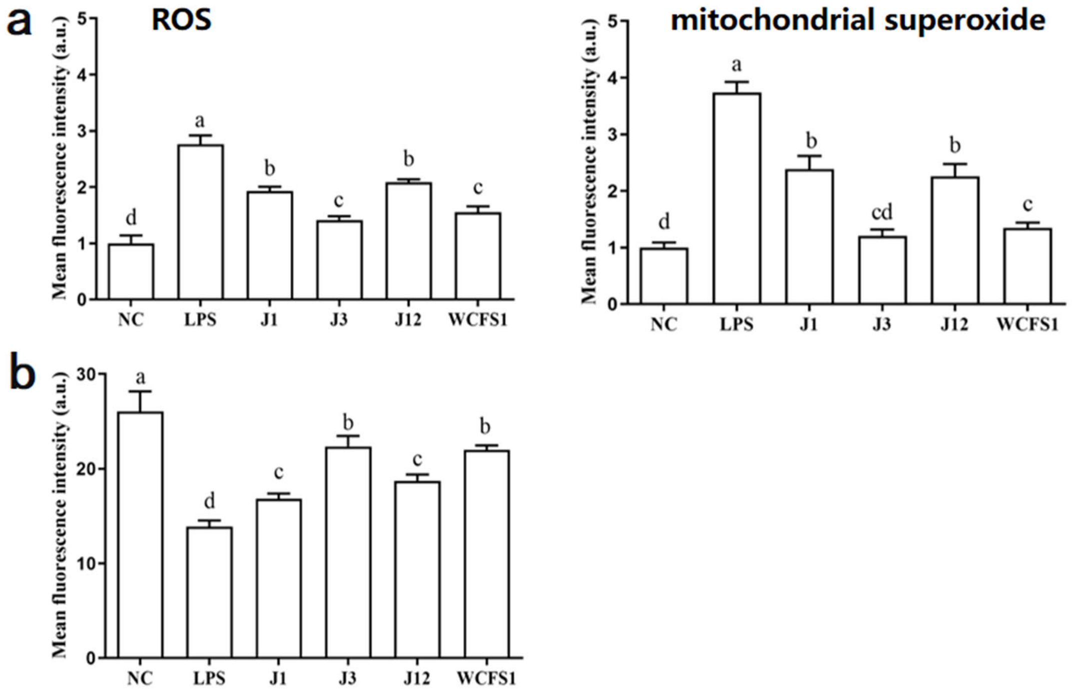

Figure S4. Effects of *Lactiplantibacillus plantarum* strains on oxidative stress in Caco-2 cells. **a.** Cellular reactive oxygen species (ROS) and mitochondrial superoxide concentrations. **b**. Mitochondrial membrane potential

2.3 The effect of *Lactiplantibacillus plantarum* on the expression of cellular antioxidant enzymes

As shown in Figure S5, compared with the control group (NC), LPS stimulation significantly reduced the mRNA expression levels of GSR, SOD1, SOD2 and GPX2 in Caco-2 cells ($P < 0.05$). *Lactiplantibacillus plantarum* strains J1, J3 and J12 all significantly reversed the downregulation of these genes ($P < 0.05$). Among these, J3 exhibited the strongest upregulatory effect on all four genes: for GSR, SOD1 and SOD2, the expression levels following J3 treatment were significantly higher than those of the other *Lactiplantibacillus plantarum* strains ($P < 0.05$); for GPX2, both J3 and J12 were significantly higher than J1 and WCFS1 ($P < 0.05$). Overall, J3 was the most effective in promoting the expression of antioxidant enzyme genes.

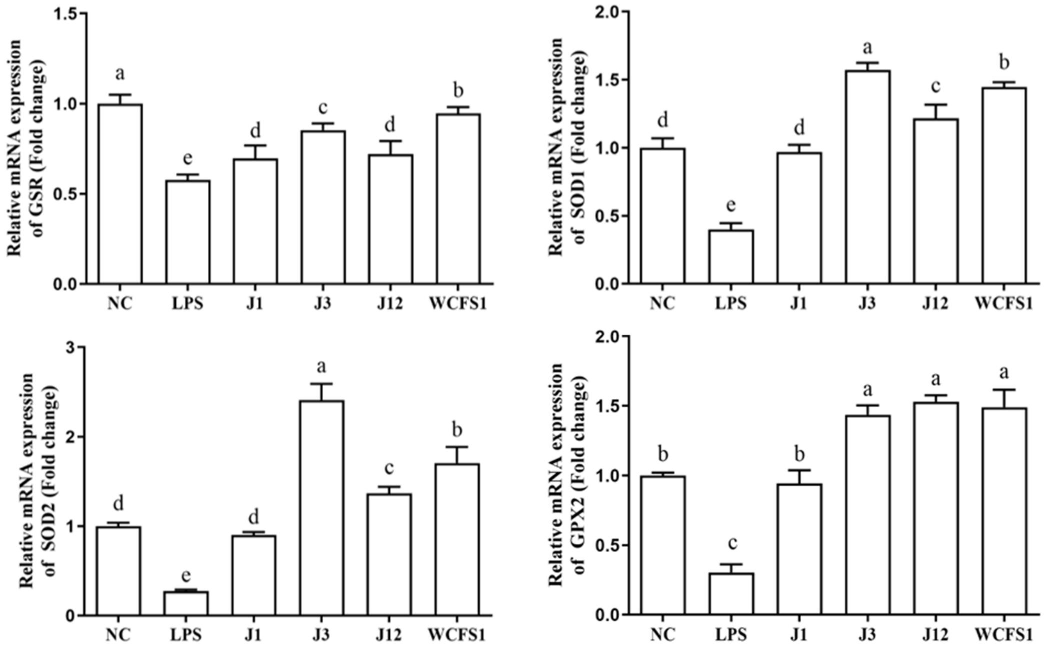

Figure S5. Effect of *Lactiplantibacillus plantarum* strains on the expression levels of antioxidant enzyme mRNA in Caco-2 cells. (GSR, SOD1, SOD2 and GPX2)

3 The effect of *Lactiplantibacillus plantarum* on the intestinal barrier of Caco-2 cells induced by LPS

3.1 The effect of *Lactiplantibacillus plantarum* on trans-epithelial electrical resistance

The effects of *Lactiplantibacillus plantarum* on LPS-induced trans-epithelial electrical resistance (TEER) in Caco-2 cells are shown in Figure S6. Using the blank group (NC) as a control, LPS stimulation significantly reduced TEER in Caco-2 cells ($P < 0.05$), whilst *Lactiplantibacillus plantarum* strains J1, J3 and J12 all significantly increased TEER in Caco-2 cells ($P < 0.05$). Compared with the control strain WCFS1, treatment with *Lactiplantibacillus plantarum* J3 did not result in any significant difference in TEER in LPS-stimulated Caco-2 cells ($P > 0.05$).

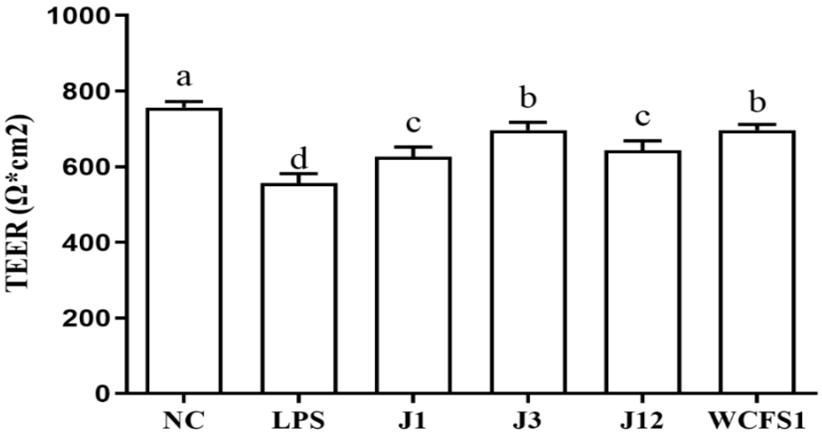

Figure S6. Effect of *Lactiplantibacillus plantarum* strains on the TEER in LPS-induced Caco-2 cells

3.2 The effect of *Lactiplantibacillus plantarum* on the expression of tight junction proteins

As shown in Figure S7, compared with the control group (NC), LPS stimulation significantly reduced the mRNA expression levels of ZO-1, Occludin and Claudin-1 in Caco-2 cells ($P < 0.05$). *Lactiplantibacillus plantarum* strains J1, J3 and J12 were all able to reverse the downregulation of these genes to varying degrees. Among them, J3 demonstrated the most effective restoration for all three tight junction protein genes: for ZO-1 and Claudin-1, expression levels following J3 treatment returned to levels not significantly different from those of the control strain WCFS1 ($P > 0.05$); for Occludin, the expression levels following J3 treatment were the highest among all tested strains. Overall, J3 demonstrated the most pronounced efficacy in repairing LPS-induced intestinal epithelial barrier damage.

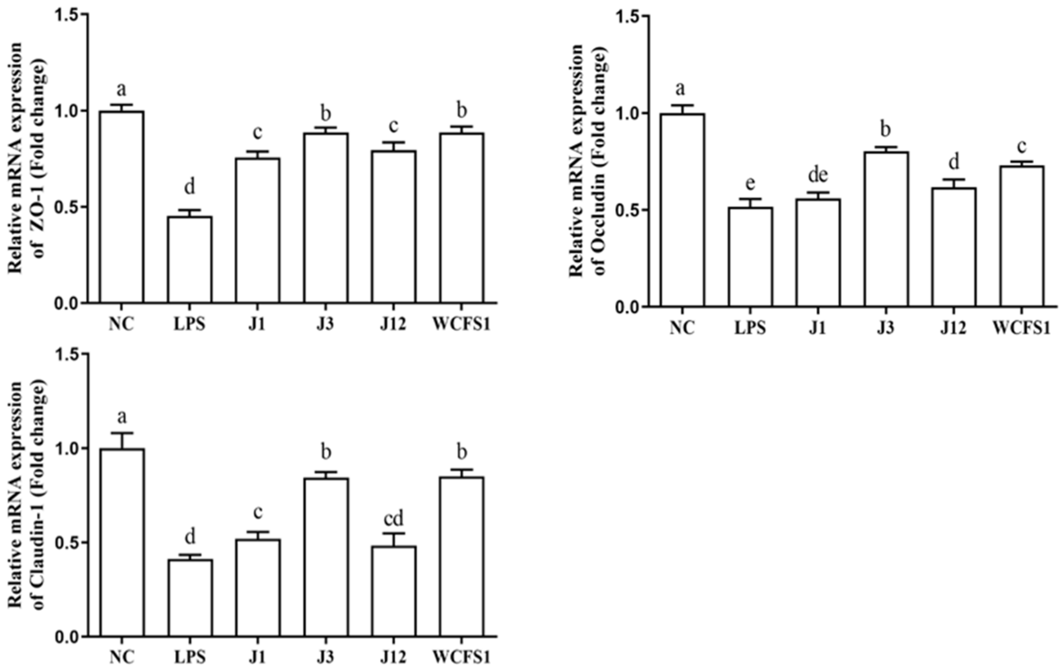

Figure S7. Effect of *Lactiplantibacillus plantarum* strains on the expression levels of tight junction protein mRNA in Caco-2 cells. (ZO-1, Occludin and Claudin-1)
