# Supplementary material for: Arctic Fox-Derived Lactiplantibacillus plantarum J3 Alleviates Colitis in Mice in Association with Strengthening of the Intestinal Barrier and Reshaping of the Intestinal Flora
Source: Life (Basel). 2026 Aug 18;16(8):1355. doi: 10.3390/life16081355 (PMC13514317; doi:10.3390/life16081355)
Supplement: Supplementary file 1 [file life-16-01355-s001.zip › Supplementary Materials S2.pdf]

Supplementary Materials S2

1. Methods for Whole-Genome Sequencing, Assembly, and Bioinformatics Analysis of the Strain

1.1. DNA Extraction

Lactiplantibacillus plantarum J3 was cultured in MRS medium, and the bacterial slurry was harvested. Total DNA was extracted from *Lactiplantibacillus plantarum* J3 using a total DNA extraction kit. After verifying the DNA quality, the samples were sent to BGI Shenzhen for sequencing.

1.2 Genome Sequencing and Annotation

A third-generation library was constructed using the standard PacBio Template Prep Kit 1.0 library preparation protocol (20 kb Template Preparation Using BluePippin Size Selection); a second-generation library was constructed using the standard Illumina TruSeq Nano DNA LT library preparation protocol (Illumina TruSeq DNA Sample Preparation Guide). Genome sequencing of *Lactiplantibacillus plantarum* J3 was performed using a combination of PacBio Sequel and Illumina NovaSeq sequencing platforms. After obtaining the raw data, quality control was performed. The primary quality control criteria for single-molecule sequencing data were: removal of adapter sequences; and a minimum polymerase read length of 50 bp. The primary quality control criteria for next-generation sequencing data were: removal of low-quality reads; removal of adapter-contaminated reads; and removal of reads containing Ns. After obtaining clean reads, Unicycler software was used to assemble the next-generation and third-generation (PacBio) data, yielding the assembly results. Prokka software was used to predict coding and non-coding genes in the newly sequenced genome. Predicted genes were aligned against GO, KEGG, COG, NR, and Swiss-Prot using BLAST (blastp, e-value ≤ 1e-5); the highest-scoring alignment result (default identity ≥ 40%, coverage ≥ 40%) from the BLAST output was annotated. A genomic map was constructed using Circlize software.

1.3 Comparative Genomic Analysis

First, the CD-HIT software was used to perform cluster analysis on the protein sequences of *Lactiplantibacillus plantarum* J3 (identity > 40%, length > 70%), identifying core and pan-genes and

generating the corresponding Venn diagrams. Extract CoreGene sequences from the whole-genome sequence of the strain. After MAFFT alignment, construct a Neighbor-Joining (NJ) phylogenetic tree using MEGA 7.0. Perform a colinearity analysis of *Lactiplantibacillus plantarum* J3 and other homologous strains using MAUVE.

2. Results of the Whole-Genome Analysis of *Lactiplantibacillus plantarum* J3

2.1 Sequencing and Assembly Results

After evaluating the quality of the sequencing data, a preliminary genome assembly of *Lactiplantibacillus plantarum* J3 was obtained, as shown in Table S1: one circular chromosome with a total length of 3,142,626 bp and a GC content of 44.75%; 5 plasmids with total lengths of 61,202, 52,615, 34,204, 8,676, and 8,292 bp, with GC contents of 39.95%, 38.94%, 40.78%, 35.96%, and 35.23%, respectively.

Table S1. Preliminary genome assembly results of L. plantarum J3.

seq	Total length (bp)	GC (%)
chr	3142626	44.75%
plas1	61202	39.95%
plas2	52615	38.94%
plas3	34204	40.78%
plas4	8676	35.96%
plas5	8292	35.23%

2.2 Distribution of Coding Gene Lengths

As shown in Table S2, the total length of the *Lactiplantibacillus plantarum* J3 genome is 3,307,615 bp, with a GC content of 44.48%; A total of 3,224 genes were predicted, with coding regions spanning 2,804,355 bp and a GC content of 45.64%. Coding regions account for 84.78% of the entire genome, with an average gene length of 870 bp. The largest number of genes fall within the 300–1,000 bp range, while the smallest number are under 100 bp.

Table S2. General feathures of L. plantarum J3.

Feather	*Lactiplantibacillus plantarum* J3
Genome Size (bp)	3307615
GC Content (%)	44.48
Gene Number	3224
Coding Gene Length:	2804355
% of Genome (Genes):	84.78
Coding Gene Average Length:	870
tRNA	69
rRNA	16
ncRNA	1
CDS	3138
CRISPR Arrays	2
Prophage	4

2.3 Repeat Sequences

Repeat sequences are identical or complementary fragments found at different locations in the genome, including scattered repeats and tandem repeats. The RepeatMasker software was used to predict scattered repeats, and TRF (Tandem Repeats Finder) was used to identify tandem repeats. As shown in Table S3, there were a total of 27 scattered repeats, including 1 long terminal repeat (LTR), 1 DNA transposon, 18 long interspersed elements (LINE), and 4 short interspersed elements (SINE). RC: rolling circle; As shown in Table S4, there were 98 tandem repeats (TRF), 84 minisatellite DNA sequences, and 1 microsatellite DNA sequence.

Table S3. Scattered repeat sequence results.

Type	Number (#)	Total Length(bp)	In Genome (%)	Average length(bp)
LTR	1	62	0.0019%	62
DNA	4	289	0.0087%	72
LINE	18	1188	0.0359%	66
SINE	4	257	0.0078%	64
RC	0	0	0	0
Unknown	0	0	0	0
Total	27	1796	0.0543%	67

Table S4. Tandem repeat sequence results.

Type	Number (#)	Repeat Size(bp)	Total Length(bp)	In Genome (%)
TRF	98	6~483	20140	0.61
Minisatellite DNA	84	9~86	13350	0.40
Microsatellite DNA	1	6	94	0.00

2.4 Genomic Islands

Genomic islands (GIs) are associated with organismal adaptation and represent genomic segments formed by the horizontal transfer of bacterial, phage, or plasmid genes into a microbial genome. As shown in Figure S1, *Lactiplantibacillus plantarum* J3 contains 11 chromosomal GIs and 1 plasmid GI, with a total GI length of 227,801 bp and an average GI length of 18,983 bp.

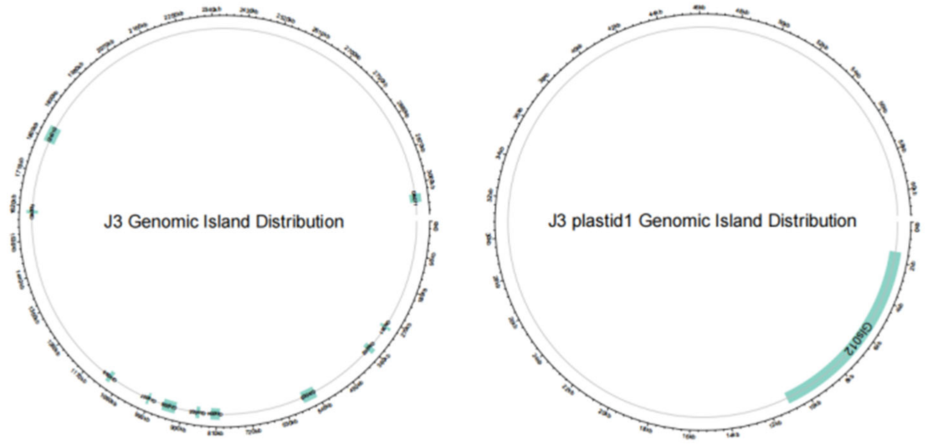

Figure S1. Genomics Islands of L. plantarum J3.

2.5 Genomic Functional Annotation

2.5.1 Functional Annotation in the NR Database

In the NR database, *Lactiplantibacillus plantarum* J3 has a total of 3,114 annotated coding genes. Of these, 486 are annotated to Lactobacillus, 2,516 to *Lactiplantibacillus plantarum*, 9 to Lactobacillus pentosus, 6 to Lactobacillus paracasei, 5 to Lactobacillus paracasei subsp. casei, and 6 to Lactobacillus fermentum.

2.5.2 GO Function Comments

Figure S2 shows the GO functional annotation diagram for *Lactiplantibacillus plantarum* J3. As shown in the figure, a total of 1,596 genes have been functionally annotated. within the Biological Process category, Metabolic Process and Cellular Process received the most annotations; within the Cellular Component category, Membrane & Membrane Part received the most annotations; and within the Molecular Function category, Catalytic Activity and Binding received the most

annotations.

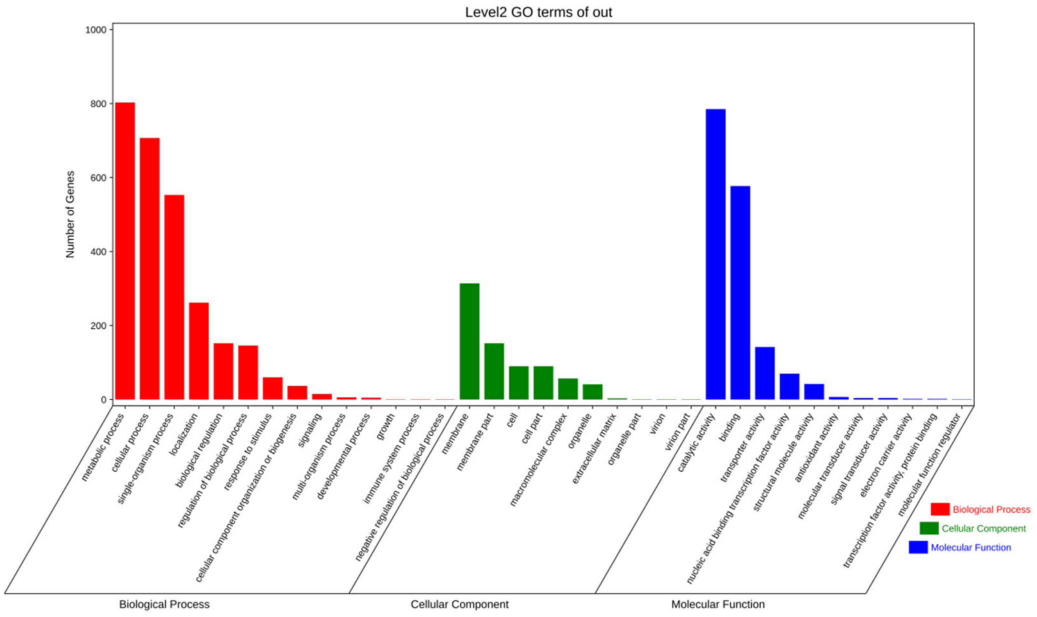

Figure S2. GO functional annotation of L. plantarum J3.

2.5.3 KEGG Functional Annotation

Figure S3 shows the KEGG functional annotation map for *Lactiplantibacillus plantarum* J3. As shown in the figure, a total of 1,528 coding genes of *Lactiplantibacillus plantarum* J3 have been annotated in the KEGG database. In the KEGG second-level classification, the strain is most heavily represented in the "Global and overview maps" pathways, followed by Carbohydrate metabolism, Amino acid metabolism, Nucleotide metabolism, Metabolism of cofactors and vitamins, Energy metabolism, and Lipid metabolism.

Within the carbohydrate category of interest, there are 40 genes involved in glycolysis and gluconeogenesis, 12 genes in the TCA cycle, 22 genes in the pentose phosphate pathway, 6 genes in pentose and glucuronate conversion, 26 genes in fructose and mannose metabolism, 34 genes in galactose metabolism, and 3 genes in ascorbic acid and aldonic acid metabolism. Among these, the 40 genes in the glycolysis and glucose synthesis pathways are shown in Figure S4, Genes include fructose-1,6-diphosphate aldolase, lactate dehydrogenase, β-galactosidase, phosphoglycerate kinase, type I glyceraldehyde-3-phosphate dehydrogenase, NADPH:quinone reductase, and 6-

phosphoglucuronidase, among others.

In the lipid metabolism category, there are 21 genes involved in fatty acid synthesis, 4 genes involved in fatty acid degradation, 2 genes involved in ketone body synthesis and degradation, and 4 genes involved in secondary bile acid biosynthesis. The fatty acid synthesis pathway is shown in Figure S5, with genes including acyl-ACP sulfatase, NAD(P)-dependent dehydrogenase, 2-nitropropane oxygenase, the biotin carboxyl carrier subunit of acetyl-CoA carboxylase, acetaldehyde dehydrogenase, and ethanol dehydrogenase, among others.

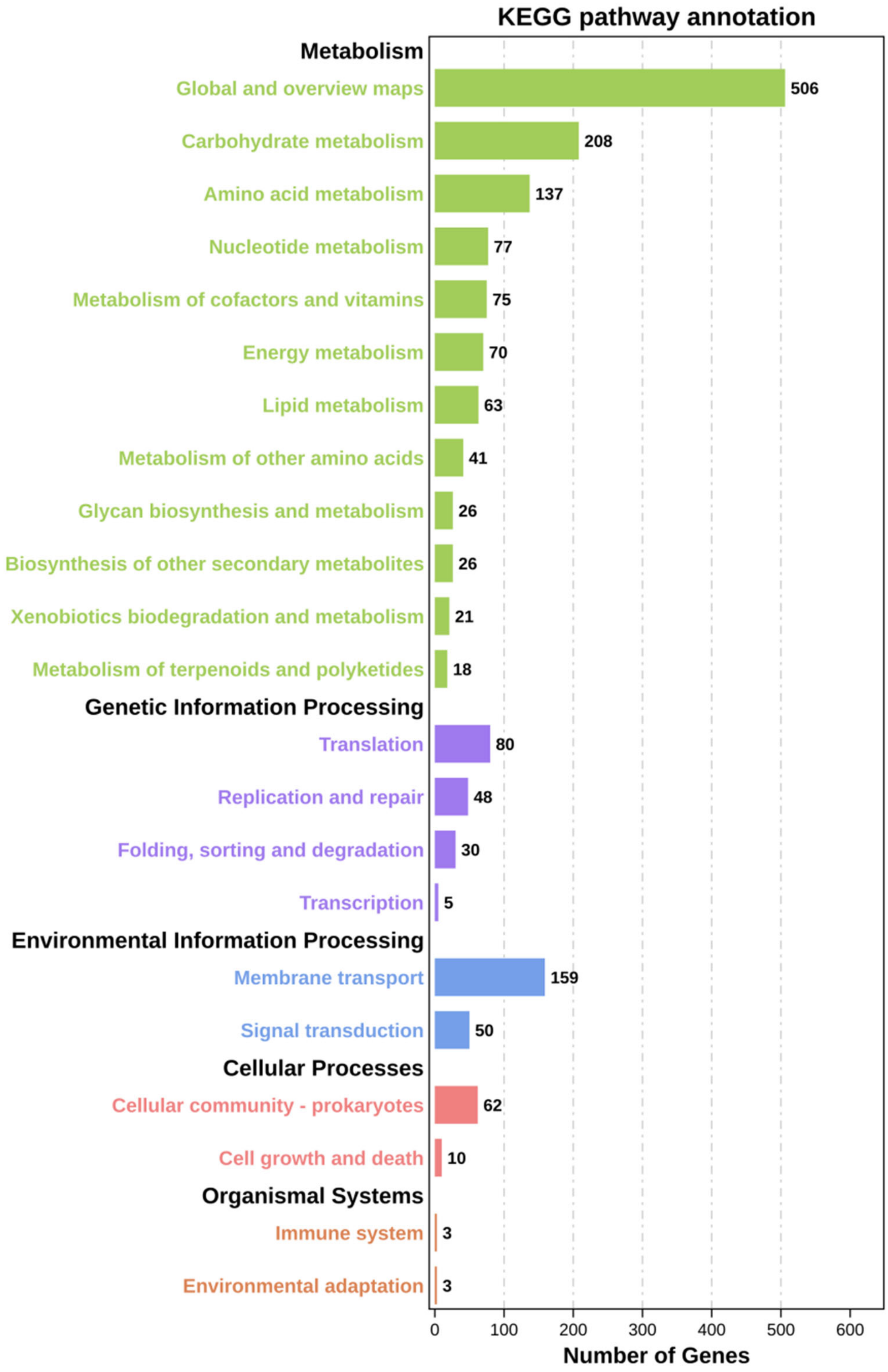

Figure S3. KEGG functional classification map of L. plantarum J3.

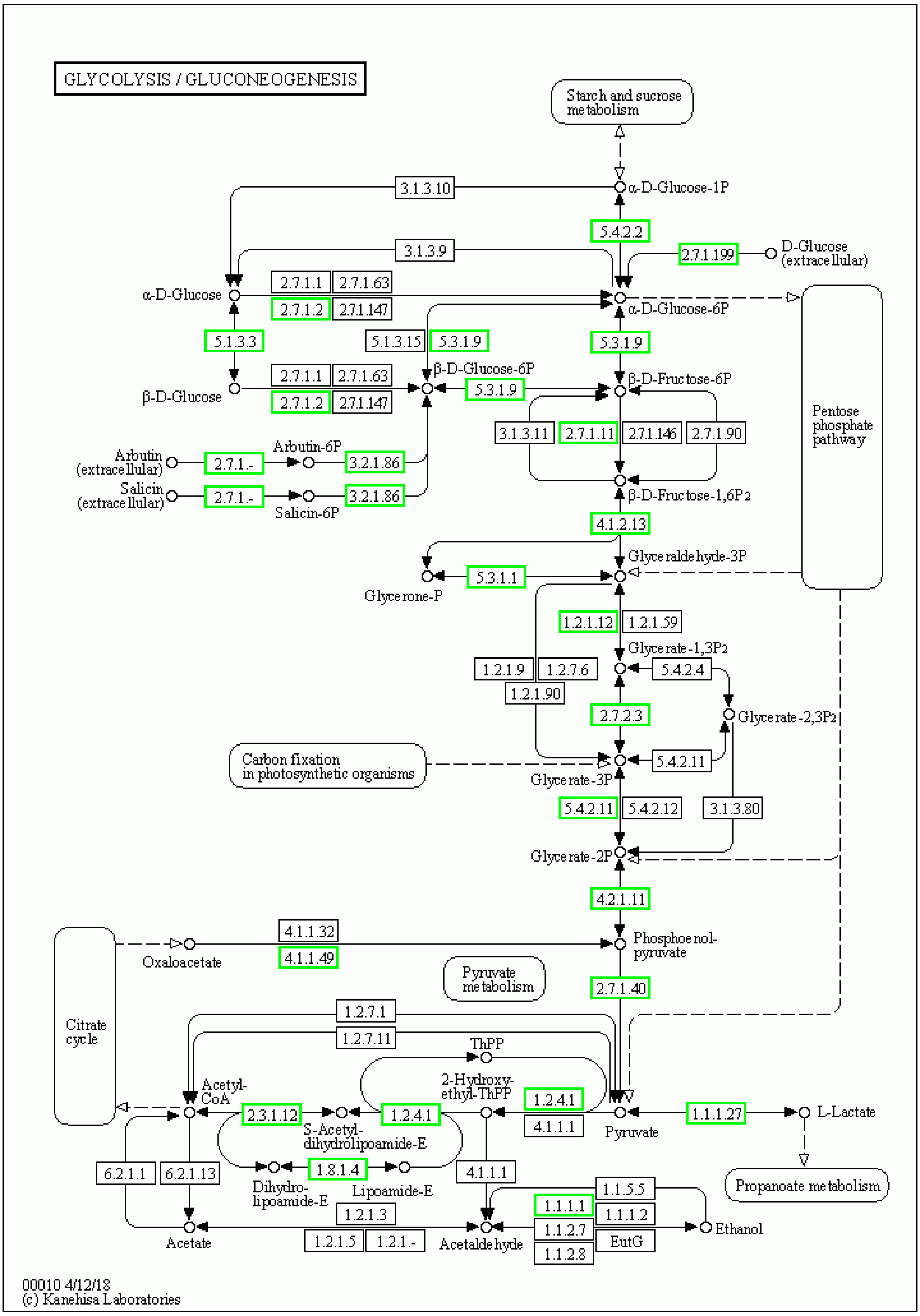

Figure S4. Glycolysis / Gluconeogenesis of KEGG functional classification map of L. plantarum J3.

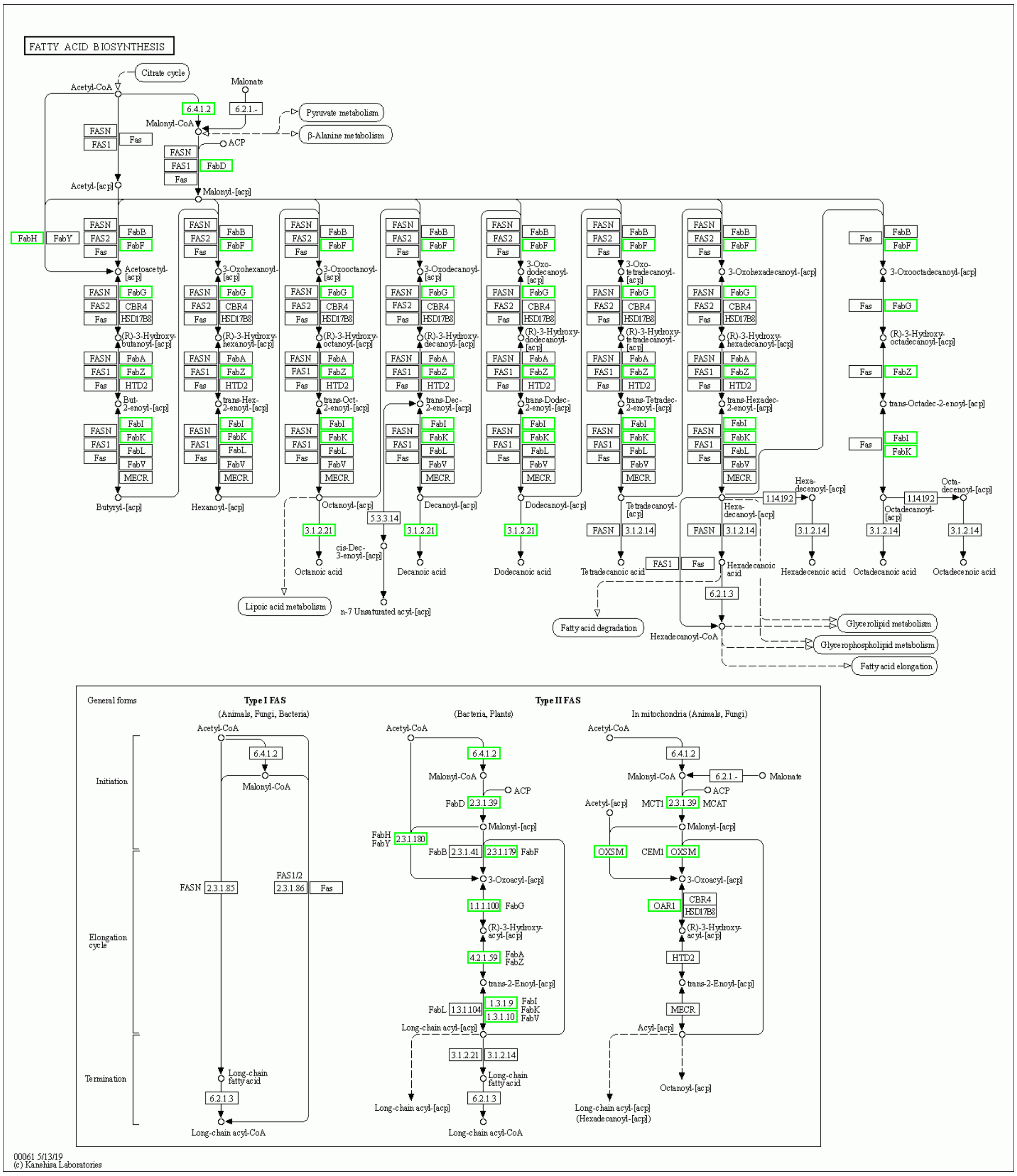

Figure S5. Fatty acid biosynthesis of KEGG functional classification map of L. plantarum J3.

2.5.4 COG Gene Bank Annotation

COG (Cluster of Orthologous Groups of proteins) assigns a COG to groups of similar protein sequences to infer their functions. Figure S6 shows the COG functional annotation diagram for *Lactiplantibacillus plantarum* J3. As shown in the figure, a total of 2,382 coding genes from *Lactiplantibacillus plantarum* J3 are annotated into 22 COG functional categories in the COG database. Among these, the functional categories with the highest number of entries are Transcription (266), Carbohydrate Transport and Metabolism (244), General Function Prediction (243), Amino Acid

Transport and Metabolism (236), and Ribosomal Structure and Biogenesis (209). These categories are followed by Cell Wall/Membrane/Envelope Biogenesis (142), Energy Production and Conversion (113) , inorganic ion transport and metabolism (130), translation, replication, recombination, and repair (116), signal transduction mechanisms (116), coenzyme transport and metabolism (115), nucleotide transport and metabolism (107), and lipid transport and metabolism (103). Additionally, the list includes post-translational modification, protein transport (83), transposons (78), defense mechanisms (78), cell cycle control, cell division, and chromosome segregation (39), biosynthesis, transport, and catabolism of secondary metabolites (33), intracellular transport, secretion, and vesicular transport (17), cell motility (17), and extracellular structures (3), among others.

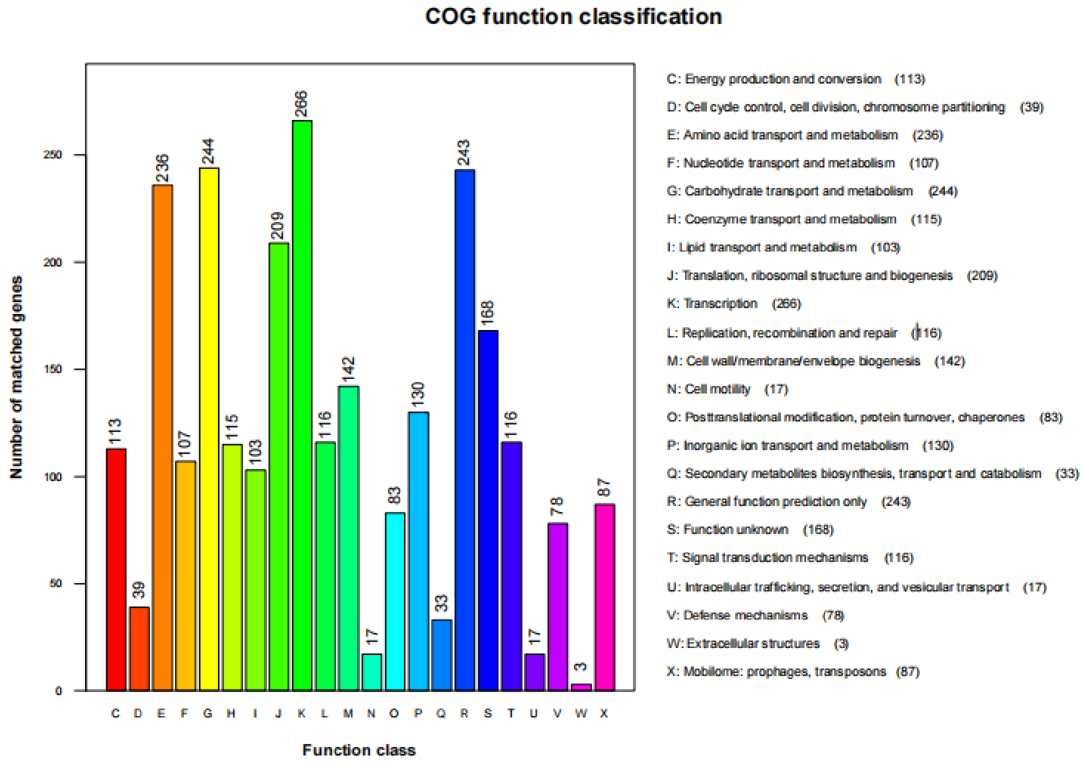

Figure S6. COG functional annotation of L. plantarum J3.

2.5.5 Functional annotation of carbohydrate-active enzymes (CAZy)

In the functional annotation of CAZy in *Lactiplantibacillus plantarum* J3, a total of 77 protein-coding genes were annotated, including 49 genes for glycosidases (GHs), 17 genes for glycosyltransferases (GTs), 3 genes for carbonyl esterases (CEs), 1 gene for oxidoreductases (AAs),

and 9 genes for carbohydrate-binding domains (CBMs).

2.6 Circular Genome Map

Based on the assembled genomic data of *Lactiplantibacillus plantarum* J3 and gene prediction results, a draft genome map of *Lactiplantibacillus plantarum* J3 was constructed (see Figure S7). The genomic structure is illustrated using a circular genome map, as shown in Figure S8. The *Lactiplantibacillus plantarum* J3 genome contains five circular plasmids: Plasmid 1 is 61,202 bp in length with a GC content of 39.95%; Plasmid 2 is 52,615 bp in length with a GC content of 38.94%; Plasmid 3 is 34,204 bp in length with a GC content of 40.78%; Plasmid 4 is 8,676 bp in length with a GC content of 35.96%; and Plasmid 5 is 8,292 bp in length with a GC content of 35.22%.

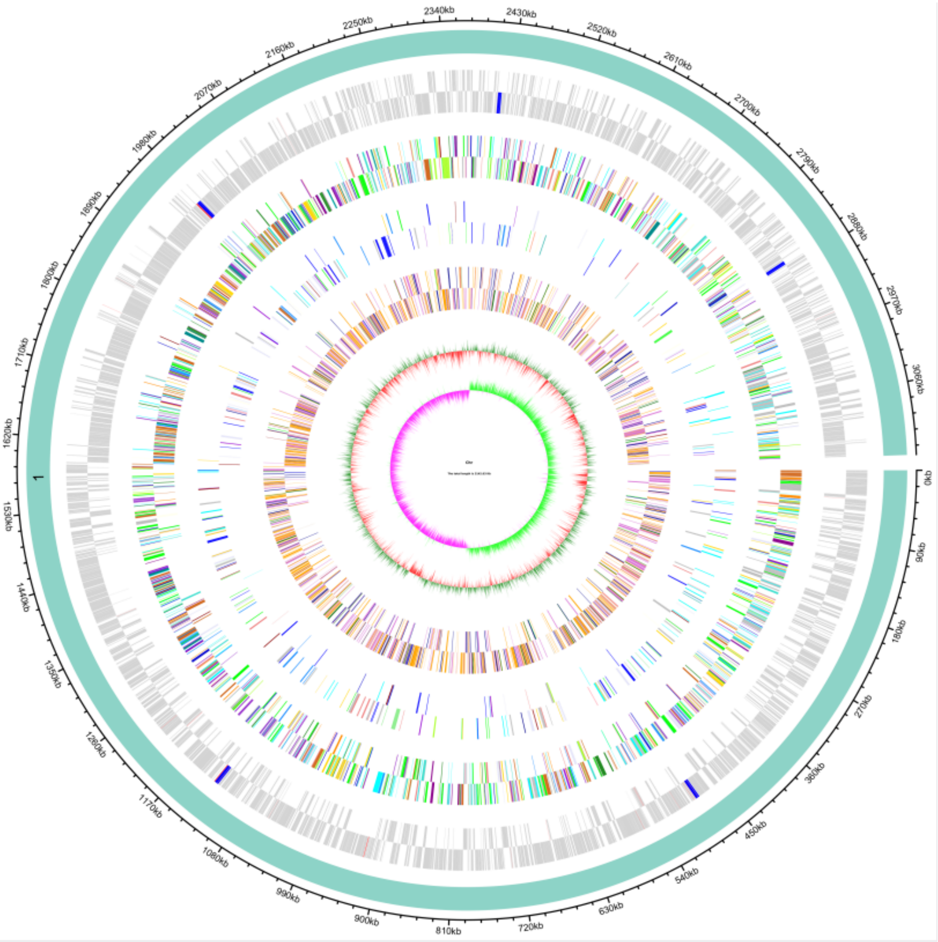

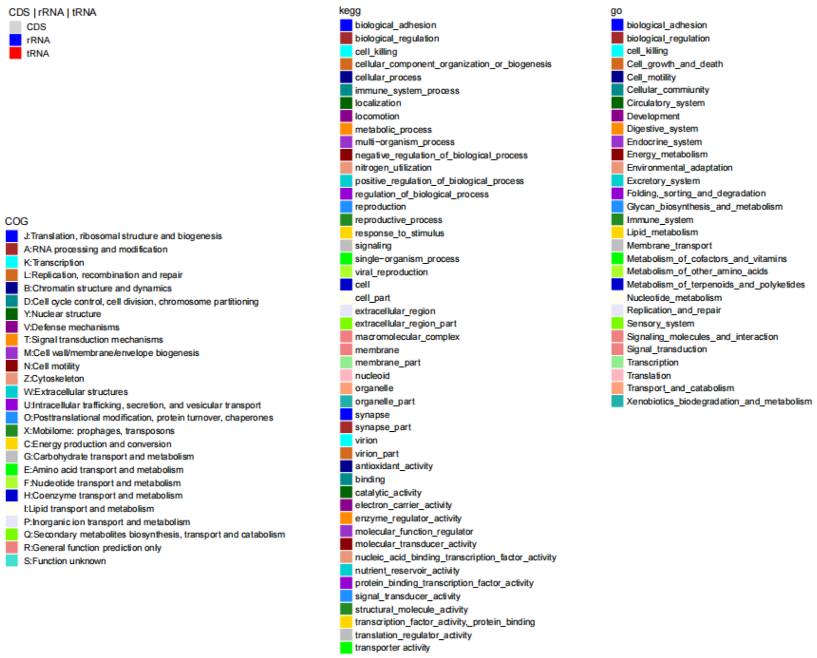

Figure S7. Genome drawing of L. plantarum J3.

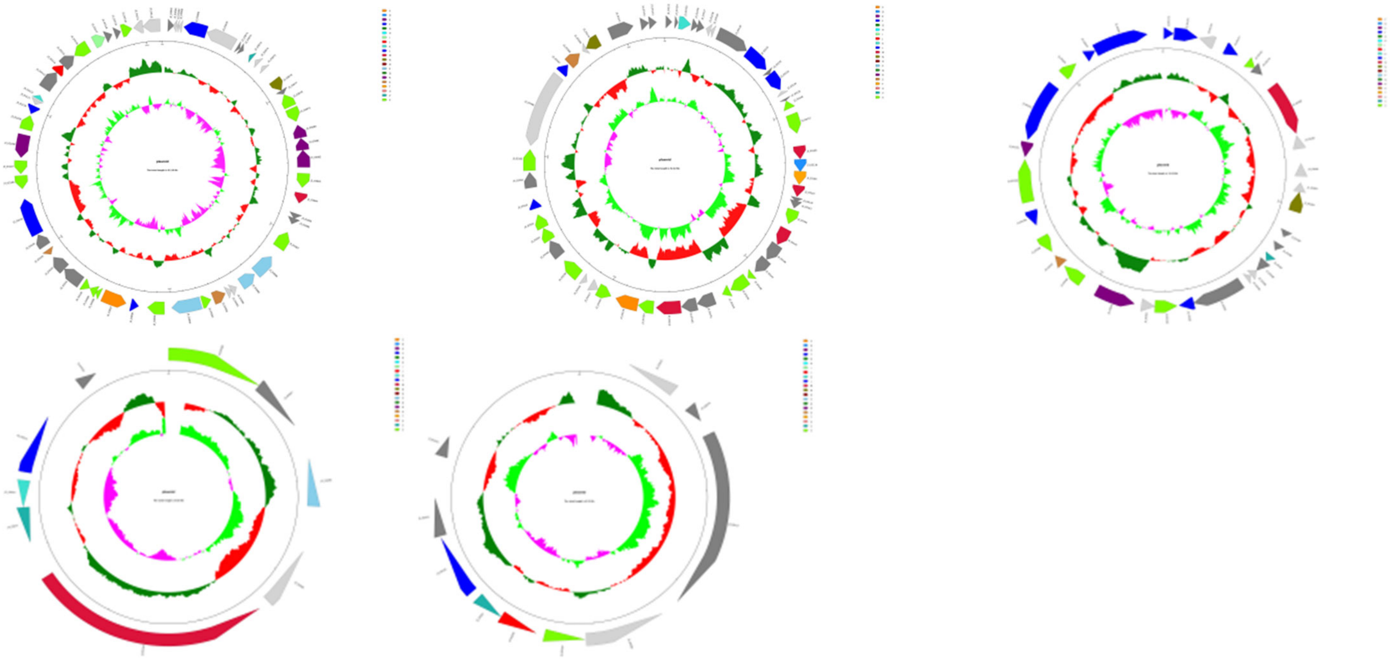

Figure S8. Circular plasmid maps of L. plantarum J3.

2.7 Core Genome and Pan-genome

In this study, we selected 15 strains of *Lactiplantibacillus plantarum* (J3, 10CH, 16, 5-2, ATCC_14917, CAUH2, EM, JDM1, KLDS1.0391, LP2, NCU116, P-8, ST-III, WCFS1, Y44, ZJ316) published in the NCBI database and known to possess certain probiotic effects as reference strains to analyze the differences in the core genome and pan-genome of *Lactiplantibacillus plantarum* J3. Genes shared by all strains are defined as core genes, while those not shared are classified as

dispensable genes; together, they constitute the pan-genome. Genes unique to a single strain are termed "specific genes" and serve as the primary basis for strain-specific functionality. Analysis revealed that the 16 *Lactiplantibacillus plantarum* strains share a total of 19,490 genes, comprising 4,769 pan-genes, 1,949 core genes, and 2,820 non-shared genes. The results of the genomic comparison between *Lactiplantibacillus plantarum* J3 and the other strains are shown in Figure S9. The 16 *Lactiplantibacillus plantarum* strains share 1,949 core genes, and there are significant differences in specific genes among the strains; *Lactiplantibacillus plantarum* J3 contains the highest number of specific genes, with 196.

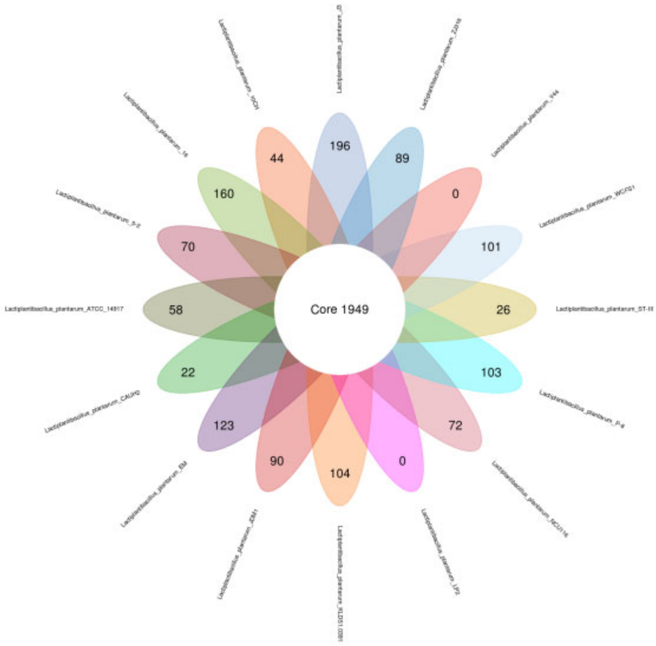

Figure S9. Venn diagram of 16 L. plantarum strains by corepan analysis.

2.8 Phylogenetic Tree Analysis

In this study, 15 strains of *Lactiplantibacillus plantarum* with known probiotic properties, as published in the NCBI database, were selected. A phylogenetic tree was constructed based on their core gene sequences to illustrate the phylogenetic relationship between *Lactiplantibacillus plantarum* J3 and other homologous strains, as shown in Figure S10. The results indicate that *Lactiplantibacillus plantarum* J3 shares the highest homology with *Lactiplantibacillus plantarum* 16, which was isolated from traditional dairy products and exhibits good probiotic activity.

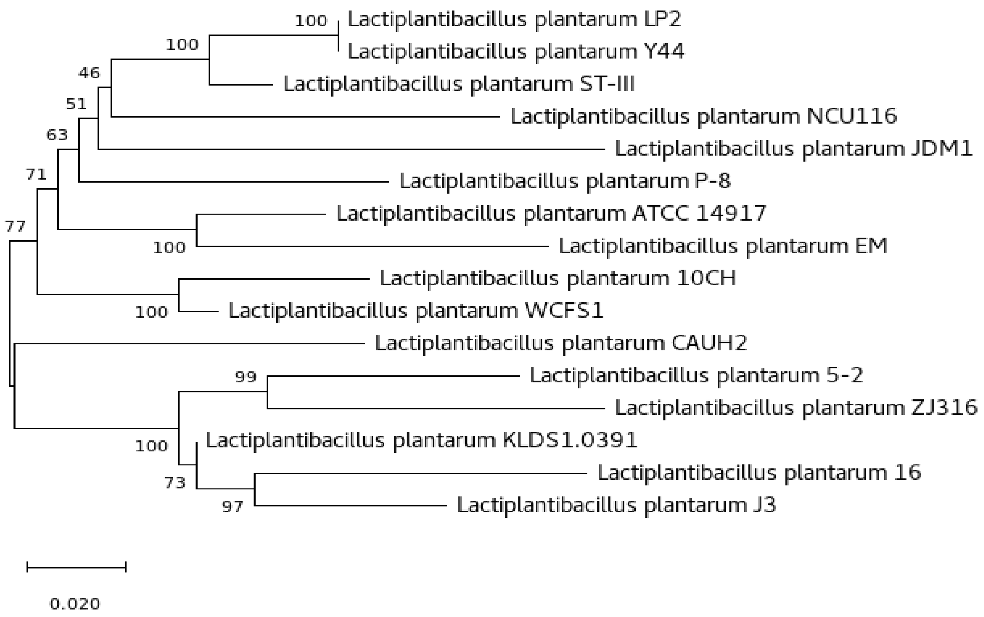

Figure S10. Phylogenetic tree based on Core Gene.

2.9 Collinearity Analysis

Lactiplantibacillus plantarum 16, 5-2, ZJ316, and KLDS1.0391 were selected as reference strains for collinearity analysis with the target strain; these strains possess excellent probiotic properties. The results are shown in Figure S11, where lines of the same color indicate similar sequences, and the height of the curves within the blocks corresponds to the similarity between the two compared sequences, representing genomic differences among the strains. As shown in the figure, *Lactiplantibacillus plantarum* J3 exhibits a good linear relationship with *Lactiplantibacillus plantarum* 16 and 5-2, with only a few translocations; compared to *Lactiplantibacillus plantarum* ZJ316, there are more translocations; and compared to *Lactiplantibacillus plantarum* KLDS1.0391, there are a large number of inversions and translocations. This suggests that *Lactiplantibacillus plantarum* J3 may have undergone genetic recombination and transfer during its evolutionary process.

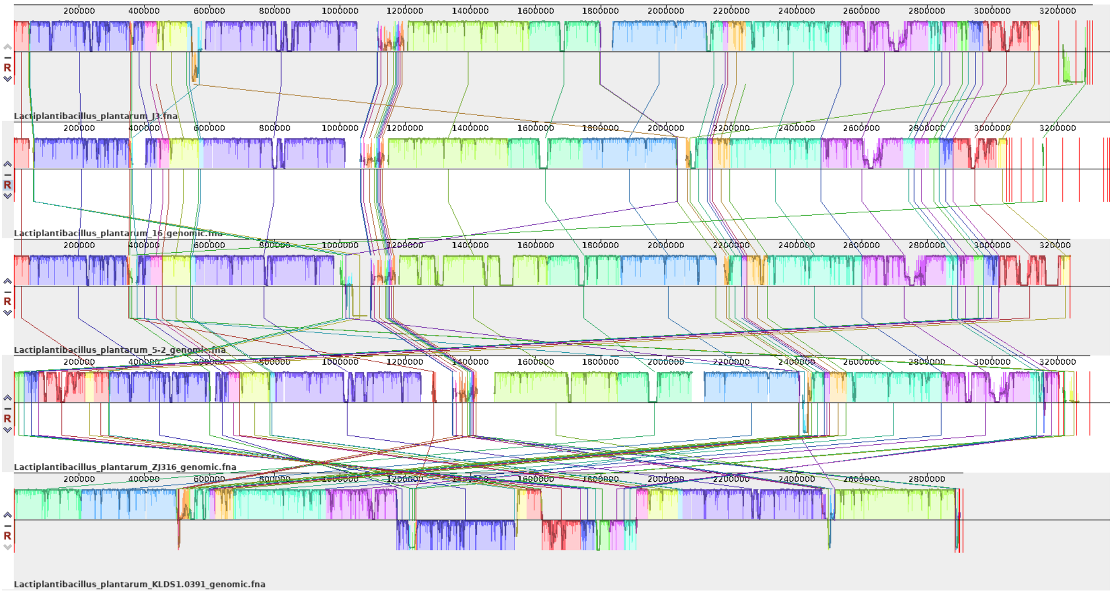

Figure S11. Synteny blocks of L. plantarum J3.

2.10 Gene Functions of *Lactiplantibacillus plantarum* J3

Figure S12 shows the KEGG functional annotation diagram for *Lactiplantibacillus plantarum* J3-specific genes. According to gene bank annotations, *Lactiplantibacillus plantarum* J3 contains a large number of genes involved in quorum sensing, carbohydrate metabolism, and other processes. As shown in the figure, *Lactiplantibacillus plantarum* J3 has a total of 196 specific genes in the KEGG database. In the KEGG second-level classification, the strain is most heavily represented in the "Cellular community - prokaryotes" pathway, followed by pathways such as Membrane transport, Global and overview maps, Carbohydrate metabolism, Biosynthesis of other secondary metabolites, Nucleotide metabolism, and Amino acid metabolism. Genes related to quorum sensing, carbohydrate, and fatty acid biosynthesis pathways in *Lactiplantibacillus plantarum* J3 are shown in Table S5. Quorum sensing-related genes include: J3_02777, J3_00349, J3_02532, J3_02531, J3_00175, J3_01398, and J3_00665; carbohydrate metabolism-related genes include: J3_01544, J3_02928, J3_02930, J3_02990, J3_03097; Genes related to fatty acid synthesis include: J3_00015, J3_00040, J3_00294, J3_02196, J3_02633, J3_03042, involving genes encoding alcohol dehydrogenase and aldehyde dehydrogenase.

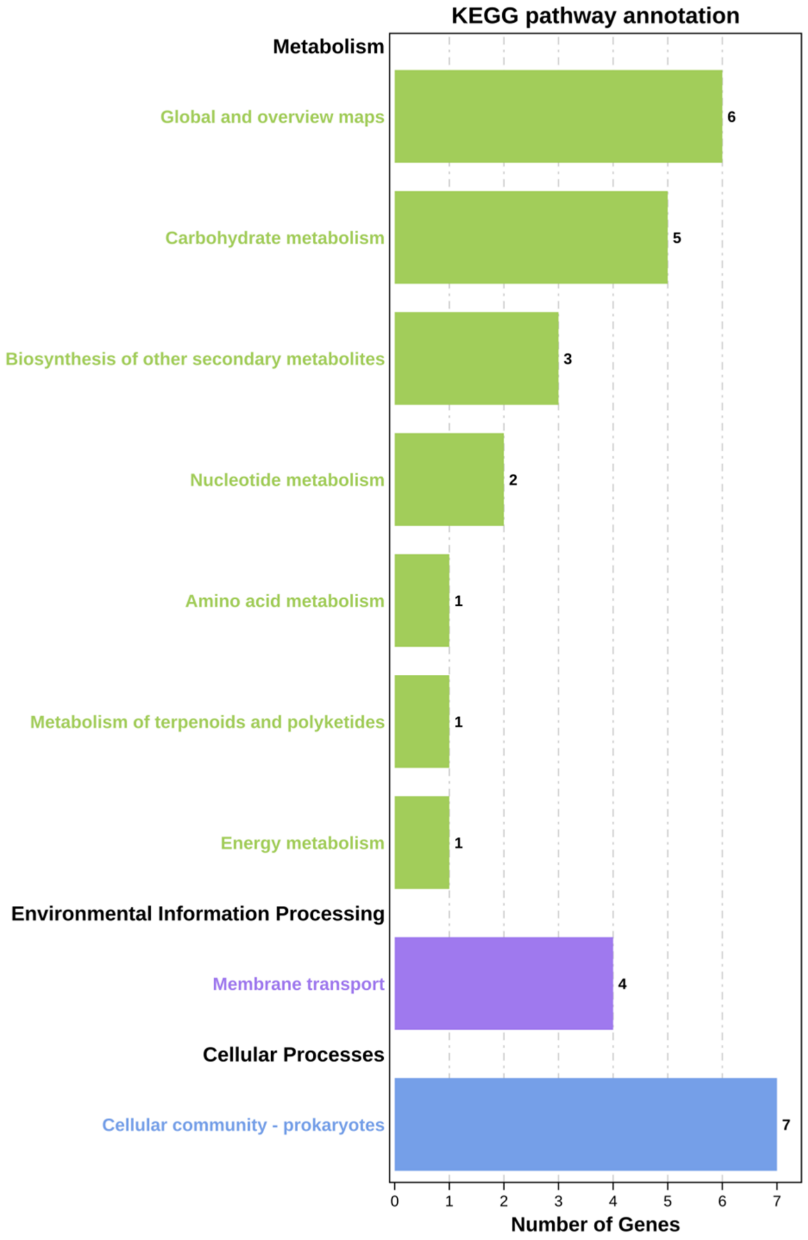

Figure S12. KEGG functional classification map of L. plantarum J3.

Table S5. Genes related to quorum sensing, carbohydrate and fatty acid metabolic pathways in L. plantarum J3.

Genetic Locus	Gene	Feature Description	
Quorum-sensing-related genes			
chr:2832994-2833290-	J3_02777	Oligopeptide Transport System ATP-Binding Protein	oppD
chr:366831-367325+	J3_00349	Contributing Factors to ABC Transporters	blpB

chr:2573939-2574583-	J3_02532	Substrate-binding protein of the branched-chain amino acid ABC transporter	livK
chr:2573392-2573886-	J3_02531	Substrate-binding protein of the branched-chain amino acid ABC transporter	livK
chr:195601-195825-	J3_00175	Peptide/Nickel Transport System Substrate-Binding Protein	ABC.PE.S
chr:1441481-1441906+	J3_01398	O-aminobenzoate synthase component II	trpG
chr:666614-667090+	J3_00665	Ribosylhomocysteine lyase LuxS	luxS
Genes related to carbohydrate metabolism			
chr:1577004-1577180+	J3_01544	Ribulose-5-phosphate 2-dehydrogenase	E1.1.1.137
chr:2987409-2988584-	J3_02928	β-galactosidase	-
chr:2989974-2990885-	J3_02930	β-galactosidase	-
chr:3063817-3065739-	J3_02990	Ketone transferase	-
plas1:33115-34794-	J3_03097	D-lactate dehydrogenase	dld
Genes involved in fatty acid synthesis			

chr:17432-18670+	J3_00015	Hemaldhyde dehydrogenase	proA
chr:47223-48650+	J3_00040	Pine aldehyde dehydrogenase	-
chr:312921-314288-	J3_00294	Acetaldehyde dehydrogenase	-
chr:2206526-2207587-	J3_02196	Aspartate semialdehyde dehydrogenase	asd
chr:2676103-2677515+	J3_02633	Succinaldehyde dehydrogenase (NAD(P)+-dependent)	gabD
chr:3116941-3119544-	J3_03042	Acetaldehyde dehydrogenase/Alcohol dehydrogenase	adhE

2.11 Genomic Safety Assessment of *Lactiplantibacillus plantarum* J3

2.11.1 Methods

To evaluate the biosafety of strain J3 at the genomic level, we followed the strategy described by Chokesajjawatee et al. (2020) [20] for whole-genome-based safety evaluation of L. plantarum. The complete genome sequence (chromosome and five plasmids) of J3 was screened for genes associated with virulence factors, biogenic amine production, and antimicrobial resistance using the following publicly available databases and tools:

(1) Virulence factor screening: The genome was searched against the Virulence Factor Database (VFDB, http://www.mgc.ac.cn/VFs/). A gene was considered a potential virulence factor if it met the criteria of >60% similarity and >60% coverage, with an E-value < $1e^{-10}$, following the less stringent criteria used in Chokesajjawatee et al. (2020) [20] to avoid missing potential hits. In addition, the KEGG BlastKOALA tool (https://www.kegg.jp/blastkoala/) was used to identify genes annotated under the "Bacterial toxins" category (KEGG map ko02042). Manual inspection of the COG and KEGG annotation results (Sections 2.5.3 and 2.5.4 of this Supplementary Material) was also performed using keywords including "hemolysin," "toxin," "virulence," "adhesin," and "invasin."

(2) Biogenic amine production gene screening: The genome was searched against the KEGG database using BlastKOALA for genes encoding key enzymes involved in biogenic amine biosynthesis, including histidine decarboxylase (EC 4.1.1.22), tyrosine decarboxylase (EC 4.1.1.25),

ornithine decarboxylase (EC 4.1.1.17), lysine decarboxylase (EC 4.1.1.18), arginine decarboxylase (EC 4.1.1.19), and agmatinase (EC 3.5.3.11), following the pathway list provided in Table 6 of Chokesajjawatee et al. (2020) [20]. The presence of any of these genes was interpreted as potential for biogenic amine production.

(3) Antimicrobial resistance gene screening: The genome was analyzed using the Comprehensive Antibiotic Resistance Database (CARD, https://card.mcmaster.ca/) with the Resistance Gene Identifier (RGI) tool under the "Perfect and Strict" hit criteria to identify acquired antimicrobial resistance genes. In addition, the KEGG BlastKOALA output was inspected under the "Antimicrobial resistance genes" category (KEGG Brite ko01504).

2.11.2 Results

(1) Virulence factors: No genes encoding known major virulence factors or bacterial toxins were identified in the J3 genome under the specified criteria. Manual inspection of the COG and KEGG annotation results using keywords related to virulence did not reveal any hemolysin, adhesin, or invasin genes. The results indicate that J3 lacks the genetic determinants typically associated with pathogenicity.

(2) Biogenic amine production: No genes encoding histidine decarboxylase, tyrosine decarboxylase, ornithine decarboxylase, lysine decarboxylase, arginine decarboxylase, or agmatinase were detected in the J3 genome. This suggests that J3 has a low potential for biogenic amine production.

(3) Antimicrobial resistance: CARD RGI analysis under "Perfect and Strict" criteria returned no hits for acquired antimicrobial resistance genes. Inspection of the KEGG Brite ko01504 category also revealed no known antimicrobial resistance determinants in the J3 genome. These findings suggest that J3 does not carry transferable antibiotic resistance genes.

2.11.3 Summary

In summary, the genomic safety assessment of L. plantarum J3 revealed no obvious virulence factors, biogenic amine production genes, or transferable antimicrobial resistance genes. These results provide preliminary genomic evidence supporting the biosafety of J3 as a probiotic candidate. However, as this assessment is based on in silico prediction, complementary phenotypic assays (e.g., hemolytic activity and antibiotic susceptibility testing) are warranted to fully establish its safety profile.
