# Supplementary material for: Arctic Fox-Derived Lactiplantibacillus plantarum J3 Alleviates Colitis in Mice in Association with Strengthening of the Intestinal Barrier and Reshaping of the Intestinal Flora
Source: Life (Basel). 2026 Aug 18;16(8):1355. doi: 10.3390/life16081355 (PMC13514317; doi:10.3390/life16081355)
Supplement: Supplementary file 1 [file life-16-01355-s001.zip › Supplementary Materials S3.pdf]

Detailed Supplement to Test Methods and Materials

Table S1. Design of RT-qPCR Primers for Cell Assays.

Gene	Forward primer (5′–3′)	Reverse primer (5′–3′)
ZO-1	AACTGGGCTCTTGGCTTGCTATTC	TCCAGAAGTCAGCACGGTCTCC
Occludin	AACTTCGCCTGTGGATGACTTCAG	GACCTTCCTGCTCTTCCCTTTGC
Claudin1	GCTGTGGATGTCCTGCGTGTC	GAGGATGCCAACCACCATCAAGG
SOD1	AGGGCATCATCAATTTCGAG	ACATTGCCCAAGTCTCCAAC
SOD2	TCC ACT GCA AGG AAC AAC AG	TCTTGCTGGGATCATTAGGG
GSR	AAGGGTCATATCATCGTAG	GTCTCCTGGTTCTCA ACG
GPX2	AGT CTC AAG TAT GTC CGTC	TGGGCTTCTTCTAAATCGTTC
GAPDH	AACGGATTTGGTCGTATTG	GCTCCTGGAAGATGGTGAT

Table S2. Design of RT-qPCR Primers for Mouse Experiments.

Gene	Forward primer (5′–3′)	Reverse primer (5′–3′)
ZO-1	GCGAACAGAAGGAGCGAGAAGAG	GCTTTGCGGGCTGACTGGAG
Occludin	TGGCTATGGAGGCGGCTATGG	AAGGAAGCGATGAAGCAGAAGGC
Claudin1	GCTGGGTTTCATCCTGGCTTCTC	CCTGAGCGGTCACGATGTTGTC
MUC2	TGCTGACGAGTGGTTGGTGAATG	TGATGAGGTGGCAGACAGGAGAC
COX-2	GGTGCCTGGTCTGATGATGTATGC	GGATGCTCCTGCTTGAGTATGTCG
β-actin	GGTTGTCTCCTGCGACTTCA	TGGTCCAGGGTTTCTTACTCC

Specific Parameters for SCFA Assay:

Separation was performed using an Agilent DB-FFAP capillary column (30 m × 0.25 mm × 0.25 μm) on a gas chromatograph (Agilent 7890B, Santa Clara, CA, USA) equipped with a flame ionization detector (FID) set to 250 °C. Chromatographic conditions: Injector temperature 250 °C; injection volume 1.0 μL; split ratio 5:1; carrier gas was high-purity nitrogen at a flow rate of 2.5 mL/min (for 6.5 min), then increased to 2.8 mL/min (for 2 min). Column temperature profile: Start at 75 °C, increase at a rate of 20 °C/min to 180 °C and hold for 1 min, then increase at a rate of 50 °C/min to 220 °C and hold for 1 min. The concentrations of each short-chain fatty acid were quantified based on a standard curve.

Table S3. Reagent Sources.

**Reagent**	**Source**
Penicillin-Streptomycin for Cell Culture	Beyotime Biotechnology (Shanghai, China)
LPS from Escherichia coli O55:B5 (Reagent grade)	Sigma-Aldrich (St. Louis, MO, USA)
ELISA kit（TNF-α、IL-1β、IL-6、IL-10、PGE2、MPO、LPS）	Nanjing Jiancheng Bioengineering Institute (Nanjing, Jiangsu, China)
D-Lactic Acid Test Kit (Colorimetric Method)	Nanjing Jiancheng Bioengineering Institute (Nanjing, Jiangsu, China)
MDA Assay Kit (TBA Method)	Nanjing Jiancheng Bioengineering Institute (Nanjing, Jiangsu, China)
Superoxide Dismutase (SOD) Test Kit (Hydroxylamine Method)	Nanjing Jiancheng Bioengineering Institute (Nanjing, Jiangsu, China)
Total Antioxidant Capacity (T-AOC) Assay Kit (ABTS Method) Microplate Format	Nanjing Jiancheng Bioengineering Institute (Nanjing, Jiangsu, China)
Glutathione Peroxidase (GSH-PX) Assay Kit (Colorimetric Method)	Nanjing Jiancheng Bioengineering Institute (Nanjing, Jiangsu, China)
RNase-free DNase I	Takara Bio Inc., Shiga, Japan
Reactive Oxygen Species Detection Kit	Beyotime Biotechnology (Shanghai, China)
Rhodamine 123	Beyotime Biotechnology (Shanghai, China)
MitoSOX Red	Beyotime Biotechnology (Shanghai, China)
Trizol	Beyotime Biotechnology (Shanghai, China)
PrimeScript RT-PCR Kit	Takara Bio
4-week-old male C57BL/6J mice	Liaoning Changsheng Biotechnology

PBS	Beyotime Biotechnology (Shanghai, China)
DSS, molecular weight: 36–50kDa	Beijing Coolaber Technology Co., Ltd.
SASP (HPLC ≥99%)	Beyotime Biotechnology (Shanghai, China)
GoScript™ Reverse Transcription Mix	Promega (Beijing) Biotech
QIAamp DNA kit	QIAGEN
MiSeq Reagent Kit V3（600 cycles）	Illumina
DMEM, High Glucose	Beyotime Biotechnology (Shanghai, China)
Transwell	Corning (Corning, NY, USA)
